# Widespread 3′ UTR splicing regulates expression of oncogene transcripts through multiple mechanisms

**DOI:** 10.1093/nar/gkaf700

**Published:** 2025-07-26

**Authors:** Jack J Riley, Cristina N Alexandru-Crivac, Sam Bryce-Smith, Stuart A Wilson, Ian M Sudbery

**Affiliations:** Sheffield Institute for Nucleic Acids, School of Biosciences, The University of Sheffield, Firth Court, Western Bank, Sheffield S10 2TN, United Kingdom; Sheffield Institute for Nucleic Acids, School of Biosciences, The University of Sheffield, Firth Court, Western Bank, Sheffield S10 2TN, United Kingdom; Sheffield Institute for Nucleic Acids, School of Biosciences, The University of Sheffield, Firth Court, Western Bank, Sheffield S10 2TN, United Kingdom; Sheffield Institute for Nucleic Acids, School of Biosciences, The University of Sheffield, Firth Court, Western Bank, Sheffield S10 2TN, United Kingdom; Sheffield Institute for Nucleic Acids, School of Biosciences, The University of Sheffield, Firth Court, Western Bank, Sheffield S10 2TN, United Kingdom

## Abstract

Splicing in 3′ untranslated regions (3′ UTRs) is generally expected to elicit degradation via nonsense-mediated decay (NMD) due to the presence of an exon junction complex (EJC) downstream of the stop codon. However, 3′ UTR intron (3UI)-containing transcripts are widespread and highly expressed in both normal tissues and cancers. We present a transcriptome assembly built from 7897 solid tumour and normal samples from The Cancer Genome Atlas. We identify thousands of 3UI-containing transcripts, many expressed across multiple cancer types. Expression of NMD component UPF1 negatively correlates with 3UI-splicing in normal, but not colon cancer, samples. 3UIs found exclusively within 3′ UTRs (*bona-fide* 3UIs) are not predominantly NMD-sensitizing, unlike introns found in 3′ UTRs due to the presence of an early premature termination codon (PTC). We identify 3UI-splicing that rescues the transcript from NMD. *Bona-fide* 3UI-transcripts are over-spliced in cancer samples. In colon cancer, differentially-spliced 3UI transcripts are enriched in the Wnt signalling pathway, with CTNNB1 showing the greatest increase in splicing. Manipulating Wnt signalling can further regulate 3UI-splicing of Wnt components. Our results indicate that 3′ UTR splicing is not a rare occurrence and 3UI-splicing can regulate transcript expression in multiple ways, some of which are likely to be EJC-independent.

## Graphical Abstract

**Graphical Abstract:**
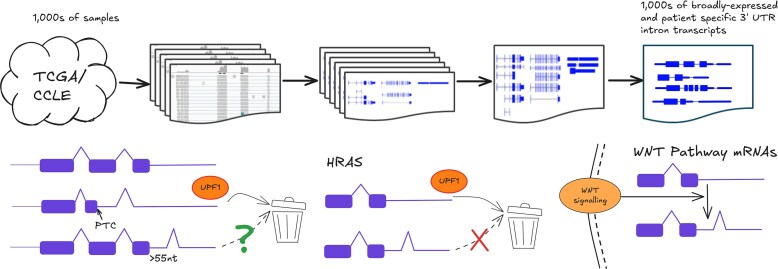

## Introduction

3′ untranslated regions (3′ UTRs) play essential roles in post-transcriptional gene regulation [1], including the regulation of transcript localization [2, 3], stability [4, 5], and translation efficiency [6, 7]. Regulation of these processes can be attributed to the presence of *cis* elements within the 3′ UTR, for example, microRNA recognition elements (MREs) and RNA-binding protein (RBP) binding motifs, which lead to the action of trans-regulators such as microRNAs (miRNAs) and RBPs. Given the important regulatory roles of the 3′ UTR, it is unsurprising that cells regulate their 3′ UTR content in cell-type and condition specific manners, with classic examples being 3′ UTR lengthening during neuronal differentiation [8–10] and shortening in cancers [11–13]. The content of the 3′ UTRs can be regulated by two main mechanisms: alternative polyadenylation (APA) and alternative splicing (AS). APA relies upon use of either distal or proximal polyadenylation sites to shorten or lengthen 3′ UTRs. AS also regulates 3′ UTR content but is not limited to the terminal sequence, instead alternate terminal exons might be used, or alternative 5′ splice sites and inclusion of earlier cassette exons might lead to the use of earlier stop codons. In this study we are interested in whether internal content can be spliced out of 3′ UTRs, which could be particularly useful in certain biological contexts where differential post-transcriptional regulation is required to impart additional functionality.

However, 3′ UTR splicing is generally seen as one signal to elicit nonsense-mediated decay (NMD). Over the last 25 years, a set of rules has emerged whereby the presence of an exon junction complex (EJC) >50–55 nt downstream of a stop codon in a translated transcript targets that transcript for degradation [14–16]. EJC-linked NMD is conserved from vertebrates to plants [16–18], although not in yeasts (which lack EJC homologues). Here sensitivity to NMD appears to be primarily determined by the presence of an aberrantly long 3′ UTR, a pathway that also operates in organisms that do have EJC-linked NMD [19]. Evidence in other organisms is contradictory. Early reports in *Drosophila melanogaster* and *Caenorhabditis elegans* [20, 21] suggested that NMD in these organisms was EJC-independent. However others showed that EJC depletion did enhance NMD in *D. melanogaster*, but that EJC only bound a subset of introns, such that not all premature termination codons (PTCs) would generate downstream EJCs [22]. Later reports demonstrated that EJC does in fact bind all *D. melanogaster* exon junctions, and so why only some introns trigger NMD when downstream of a stop codon remains a mystery [23].

Thus, transcripts with spliced 3′ UTRs have generally been considered transcriptional noise and assumed to be broadly nonfunctional. Indeed, the GENCODE project labels transcripts with stop codons >50 nt upstream of a splice site as nonprotein coding by default, unless they are the only isoform of a known protein coding gene. However, there are a limited number of specific examples where eliciting NMD to produce short half-life transcripts may be functionally beneficial. For example, multiple SRSF (SRSF1–12) transcripts contain ultra-conserved poison exons which are subject to inclusion or excision via AS to modulate NMD and dampen expression, thereby maintaining steady state SRSF protein expression [24]. Poison exon inclusion has also been shown to autoregulate expression of heterogeneous nuclear RNP proteins [25, 26]. Additionally, a 3′ UTR intron (3UI) within the Arc messenger RNA (mRNA) leads to its degradation by NMD [27]. Whilst most Arc mRNA is processed and translated upon nuclear export, it has been reported that a subset of Arc mRNA is transported to neuronal dendrites in a translationally silent state, where, upon BDNF signalling, it is rapidly translated and degraded shortly thereafter with such rapid bursts of expression and degradation potentially contributing towards long term potentiation and memory consolidation [28].

Whilst other specific examples of 3′ UTR splicing mediated regulation exist [29], they are limited to static biological contexts and do not address whether 3′ UTR splicing changes in different systems. In this study we investigate how 3′ UTR splicing impacts post-transcriptional regulation in cancer and how this differs from normal tissue. We achieve this by analysing a cohort of 7897 primary tumours and normal samples from The Cancer Genome Atlas (TCGA) as well as samples from the Cancer Cell Line Encyclopedia. Through the creation of a 3UI-centric transcript assembly we investigate whether 3′ UTR splicing triggers NMD in the same way a PTC does. Additionally, we investigate how 3′ UTR splicing regulates the composition of *cis* elements and the binding of trans-factors, and the effects of these changes on mRNA export and stability.

## Materials and methods

### Identification and quantification of 3UI expression

Raw RNAseq reads from TCGA [30] or the Cancer Cell Line Encylopedia (CCLE) [31] were downloaded in .fastq format. Read quality control was conducted with FASTQC[32] to ensure good quality base calls, GC distributions and duplication rates in a subset of samples. Reads aligned to hg38 for TCGA samples passing QC were obtained from the Genomic Data Commons (dbGaP accession phs000178.v11.p8) in BAM format. Raw reads for CCLE samples were mapped to hg38 with STAR [33] using junction annotations from ENSEMBL 85.

3UI detection was facilitated via a custom bioinformatic pipeline (www.github.com/sudlab/Cancer3UIs/pipelines/pipeline_utrons_assemble.py). Briefly, reads were first filtered by Portcullis [34] to retain only the spliced reads that are likely to be genuine; novel transcripts were then assembled with StringTie [35], retaining only transcripts representing >5% of expression from a locus (-f 0.05). Assemblies from individual samples were then merged, keeping only transcripts with a Transcripts Per Million (TPM) of >1.

Once this pipeline had been run on each cancer type individually, all assemblies were merged into a master “all TCGA” assembly or “all CCLE”, to facilitate comparison between different cancer types downstream. Merged assemblies were filtered to remove transcripts that were fragments of reference transcripts, contained entirely within introns or UTRs of reference transcripts or did not overlap any reference transcript (TCGA assembly in Supplementary Data 1, CCLE assembly available online as part of our on-line data submission at http://doi.org/10.15131/shef.data.25612722). 3UIs are then detected by comparison of either the total Ensembl annotation, the CHESS v2 annotation, or the TCGA/CCLE assemblies against a high confidence reference assembly (Ensembl85; transcript support level 1 or 2 and having an APRIS primary or alternate 1 or 2 annotation) to classify 3UIs. Briefly, each transcript in the assembly is compared to each transcript in the reference set that has a start codon in an exon of the query transcript. A 3UI transcript is identified when the transcript shares identical intron chains within the boundaries of the reference transcript's CDS, and additional introns 3′ of the reference stop codon. Identity of CHESS, TCGA, and CCLE 3UI containing transcripts and the intron locations are provided in Supplementary Data 2, 3, and 4, respectively. The complete structure of these transcripts, including the implied CDS is included in Supplementary Data 5 for TCGA transcripts, and in the online data supplement at http://doi.org/10.15131/shef.data.25612722 for CHESS and CCLE transcripts (see Appendix for description of Supplementary data files). Transcripts are classed as ‘exclusively 3′ UTR’ (e3UI) if the 3UI does not share an exon–intron or intron–exon boundary with a coding intron in any reference transcript, otherwise they were classed as CDS overlapping (co3UI). CHESS, TCGA, and CCLE e3UI locations and transcript identities are provided in Supplementary Data 6, 7, and 8 respectively (see Appendix for description). Transcripts are classified as ‘novel’ if they contain a 3UI that does not share any exon–intron or intron–exon junction with any reference transcript. Identities of novel 3UI containing transcripts and the intron locations are provided in our online data resource at http://doi.org/10.15131/shef.data.25612722. The distance of the 3UI from the stop codon is recorded as the distance from the stop codon of the matched reference transcript. Genesets were compared using trmap (https://github.com/gpertea/trmap). This pipeline can be found at www.github.com/sudlab/Cancer3UIs/pipelines/pipeline_utrons_annotate.py.

We checked for spurious assembly of 3UI transcripts by simulating 60 samples of RNAseq reads from the Ensembl 85 annotation using Polyester [36], with all transcripts simulated at 1 TPM, and passing the resulting files through the pipeline outlined above. Any gene for which a transcript with a novel 3UI was identified by this process was excluded from further analyses.

Once a pan-TCGA assembly had been obtained, samples were then quantified against the appropriate assembly using Salmon (with options –gcBias –reduceGCMemory) with an index using the full sequence of hg38 as decoy, and reads mapping to exons and junctions counted using featureCounts [37] (with options -fJOG). Complete quantifications of every sample against the TCGA assembly are available in our online data resource (see Appendix for description).

Transcripts were classed as “broadly expressed” in a given tissue where they had a TPM value > 1 and fraction expression (Transcript TPM ÷ Gene TPM) > 0.25 in at least 10% of either cancer or normal samples, and evidence of all junctions being covered by at least one read from featureCounts. The list of broadly expressed transcripts in each cancer type is available in Supplementary Data 9 (description in Appendix).

The code for this pipeline can be found at www.github.com/sudlab/Cancer3UIs/pipelines/pipeline_utrons_requant.py.

### Correlation of average 3UI PSO versus gene expression per sample

For this analysis individual event level PSO (1-PSI, as calculated by rMATS, see below) values for normal and cancerous colon tissue from TCGA were summarized to produce average 3UI PSO values for both e3UIs and co3UIs, per sample. The average 3UI PSO values were then correlated with the normalized gene expression value of every protein coding gene in the genome. Normalization of gene expression was conducted using the DESeq2 method [38]. To test the strength and significance of correlations between normalized gene expression and average 3UI PSO, Spearman’s rank correlation coefficient was calculated per gene per condition (normal e3UI, normal co3UI, cancer e3UI, cancer co3UI). *P*-values were adjusted via Benjamini–Hochberg correction. To test for the significance of correlation differences between normal and cancer samples we used the cocor package [39].

### RBP and miRNA enrichment analyses

RBP and miRNA interaction annotations were downloaded from starBase/ENCORI [40]. These were subsequently compared with a .bed file containing the locations of e3UIs using ‘GAT’ [41]. Fold change is a representation of observed motif frequency compared with expected frequency (which is generated through randomized sampling). Enrichment was considered statistically significant where adjusted *P*-values ≤ .01.

### Cell culture and treatments

Human colorectal carcinoma cell lines HCT116 and SW620 were cultured in high glucose Dulbecco’s modified Eagle’s medium (4.5 g/l) supplemented with 10% fetal calf serum and penicillin–streptomycin. H9 human embryonic stem cells were cultured in mTeSR Plus (STEMCELL Technologies). All cell lines were maintained at 37°C and 5% CO_2_ (standard conditions). For Wnt manipulation assays cells were initially grown for 24 h under standard conditions, before media was changed to RPMI 1640 medium (Gibco #11875093) supplemented with varying concentrations (0–20 μM for HCT116 and SW620; 0–11 μM for H9) of CHIR99021 (for 24 h) or various concentrations (0–20 μM for HCT116 and SW620; 0–5 μM for H9) of IWR-1 (for 48 h).

### RNA extraction, genomic DNA (gDNA) extraction, reverse transcription quantitative polymerase chain reaction (RT-qPCR), RNA sequencing (RNAseq)

RNA extraction was conducted using TRI Reagent (Sigma–Aldrich) in line with the manufacturer’s instructions. DNA removal was facilitated by TURBO DNAse (Invitrogen) treatment for 1 h, in the presence of RiboSafe RNase Inhibitor (Bioline). Reverse transcription was conducted using High-Capacity complementary DNA (cDNA) Reverse Transcription Kit (Thermo Fisher) in the presence of RiboSafe RNase Inhibitor. gDNA was extracted by lysing and incubating cells in 199 μl 1 M Tris–ethylenediaminetetraacetic acid, 0.5 μl 20% sodium dodecyl sulfate, and 0.52 μl 19 mg/ml proteinase K for 4 h at 60°C with shaking, followed by phenol-chloroform extraction and EtOH precipitation.

RT-qPCR was conducted using Quick-Load Taq 2X Master Mix (NEB). For molecular cloning applications Q5 High-Fidelity DNA polymerase (NEB) was used. RT-qPCR was conducted using SensiMix SYBR Hi-ROX 2× Master Mix (Bioline) on the Rotor-Gene Q (Qiagen). For sequencing applications RNA was extracted as previously described, quality was assessed by Qubit RNA Broad-Range Assay Kit (Thermo Fisher), mRNA library preparation and Illumina short-read (PE150) sequencing was conducted by Novogene (Cambridge, UK).

### NMD inhibition

HCT116 cells were seeded 24 h prior to siRNA transfection to achieve 30% confluency at the time of transfection. Small Interfering RNA (siRNA) duplexes against UPF1 (UPF1_1: CAGUUCCGCUCCAU UUUGAU; UPF1_2: GAUGCAGUUCCGCUCCAUU) were transfected at 30 nM using Lipofectamine RNAiMAX (Invitrogen) as per the manufacturer’s instructions. Cells were transfected in the same manner 48 h later and harvested 72 h following initial transfection. Efficiency of UPF1 knockdown was assessed by western blotting and RT-qPCR. RNA-seq experiment was conducted using siUFP1_1. Validation and follow up experiments conducted using siUPF1_2. For small molecule NMD inhibition, UPF1 inhibitor NMDI14 (Sigma–Aldrich #SML1538) was supplemented into culture media 24 h prior to RNA extraction at a final concentration of 65 μM. Cells were treated with all pairwise combinations of siRNA and inhibitor, but analysis of results showed little effect of NMDI14 either together with siUPF1 or with siDsRed, and so all siUPF1 or siDsRed transfected samples were analysed together.

### Western blotting

Twenty micrograms of protein was resolved by sodium dodecyl sulfate–polyacrylamide gel electrophoresis in a Mini-PROTEAN vertical electrophoresis cell (Bio-Rad) alongside PageRuler Prestained Protein Ladder (Thermo Scientific #26619). Protein transfer was performed onto nitrocellulose membranes using the Trans-Blot Turbo (Bio-Rad) at 25 V, 1.3 mA for 25 min. Membranes were blocked with 5% milk in TBST for 1 h at room temperature followed by incubation with 1:500 anti-UPF1 antibody (Proteintech #66898) or 1:5000 anti-Tubulin (Sigma–Aldrich #T6199) in Tris-Buffered Saline with Tween 20 (TBST) + 5% milk for 2 h at room temperature. Membranes were washed 3× with TBST for 5 min. Membranes were then incubated with 1:10 000 horseradish peroxidase-conjugated secondary antibody (Promega #W402B) in TBST + 5% milk for 1 h at room temperature. Membranes were washed 3× with TBST for 5 min. Signal was developed by ECL reagent for 30 s before the membrane was exposed using a Bio-Rad Chemidoc.

### Event- and transcript-level splicing analyses

For differential transcript usage (DTU) analysis DRIMSeq [42] together with stageR[43] was used. For siUPF1 knockdown in HCT116 cells, samples were quantified with Salmon as described above against the TCGA transcript assembly. Transcripts were only considered for DTU analyses where they had counts >10 and fraction expression >0.1 in >10% of samples. The output of DRIMSeq was filtered for 3UI-containing transcripts before *P*-value adjustment, adjusted *P*-values ≤ .05 were considered statistically significant.

rMATS-turbo [44] was used for differential splicing analysis and to calculate PSI/PSO values, using a retained intron fixed event set generated from our ‘all_TCGA’ transcript assembly. Our rMATS pipeline can be found at www.github.com/sudlab/Cancer3UIs.

For analysis of NMD inhibition, PCA analysis revealed little effect of NDMI14 on splicing, and so NMDI14 and Dimethyl Sulfoxide (DMSO)-treated samples for a particular siRNA were compared together. For analysis of TCGA RNAseq data each cancer type was analysed separately. Inputs were provided in .bam format, where ‘b1’ was set as cancer samples and ‘b2’ was set as normal samples, meaning events with IncLevelDifference < 0 are more spliced in cancer than in normal. Complete results for each cancer type are available in our online data resource. For Wnt manipulation RNAseq data we set ‘b1’ as 0 μM CHIR99021 and ‘b2’ as 20 μM CHIR99021; therefore events with IncLevelDifference > 0 are more spliced in 20 μM CHIR99021 treated. Only results produced from junction counts (not exon counts) were taken for downstream analysis. Events were considered significant where False Discovery Rate (FDR) < 0.1. Sashimi plots were generated using ‘rmats2sashimiplot’ (www.github.com/Xinglab/rmats2sashimiplot). To identify the gene sets enriched for increased splicing between colon cancer and noncancer, all 3UIs were ranked from most highest PSO increase in cancer (lowest IncLevelDifference) to highest PSO decrease in cancer (highest IncLevelDifference) and gene set enrichment analysis (GSEA) was conducted using fgsea [45]. To address whether the canonical Wnt signalling pathway was enriched in CHIR99021 treated HCT116 cells, we ranked all 3UIs from most over-spliced in 20 μM treated (highest IncLevelDifference) to most over-retained in 20 μM treated (lowest IncLevelDifference) and tested specifically for the MSigDB C5 M12752 gene set (GOBP_CANONICAL_WNT_SIGNALING PATHWAY).

### Molecular cloning

Full length CTNNB1 and HRAS 3′ UTRs were amplified using Q5 High-Fidelity DNA polymerase from HCT116 gDNA. Following DpnI digest and gel extraction, PCR products were cloned into pCI-neo (Promega) downstream of the Luciferase2 gene under the control of a CMV promoter. Q5 Site-directed mutagenesis kit (#E0554S, NEB) was used to produce intronless, 5′ss and 3′ss mutants as per the manufacturer’s instructions. Plasmids were verified by Sanger sequencing.

### Luciferase assays

HCT116 cells were seeded at 125 000 cells per well into 24-well plates. Cells were transfected after 24 h with 500 ng of Luc2-CTNNB1/HRAS and 5 ng hRluc plasmids using polyethylenimine (PEI) at a 1:3 DNA:PEI ratio. Transfected cells were cultured under standard conditions for 72 h before being washed with phosphate-buffered saline and lysed and processed using materials provided with the Dual-Luciferase Reporter Assay System Kit (Promega).

### RNA stability assays

HCT116 cells were seeded at 300 000 cells per well into six-well plates. After 48 h culture media was supplemented with 5 μg/ml actinomycin D. Cells were harvested at 0, 3, and 12 h following the addition of actinomycin D. RNA was extracted with Total RNA Purification Plus Kit (#48400; Norgen Biotek). Isoform expression was measured via qPCR following cDNA synthesis (previously described).

### Differential transcript expression analysis

Differential transcript expression analysis for UPF1 knockdown in HCT116, CHIR99021 treatment in HCT116, and nucleocytoplasmic fractionation in HCT116 [26] (GSE228810) were conducted using DESeq2. Transcript expression was estimated using Salmon as above using the complete transcript assembly from TCGA. Principal component analysis (PCA) was performed as a method of quality control. For UPF1 knockdown in HCT116 PCA indicated no effect of the small molecule inhibitor; therefore, the primary coefficient used in this analysis was siUPF1 versus siDsRed. Only transcripts with >1 read in at least four samples in the same condition were considered. ECDF (empirical cumulative distribution function) plots were generated to compare Log2FoldChange trends between subsets of transcripts. For nucleocytoplasmic fractionation data analysis a prior filtering step was conducted to ensure that only transcripts from protein coding genes were analysed, and that these had a transcript expression of >1 TPM in either the nuclear or cytoplasmic fraction. The analysis pipeline can be found at https://github.com/sudlab/Cancer3UIs/tree/main/scripts.dir/Fig2.

## Results

### A large set of highly expressed 3UI containing transcripts identified from cancer and normal RNA-seq data

**Figure 1.**
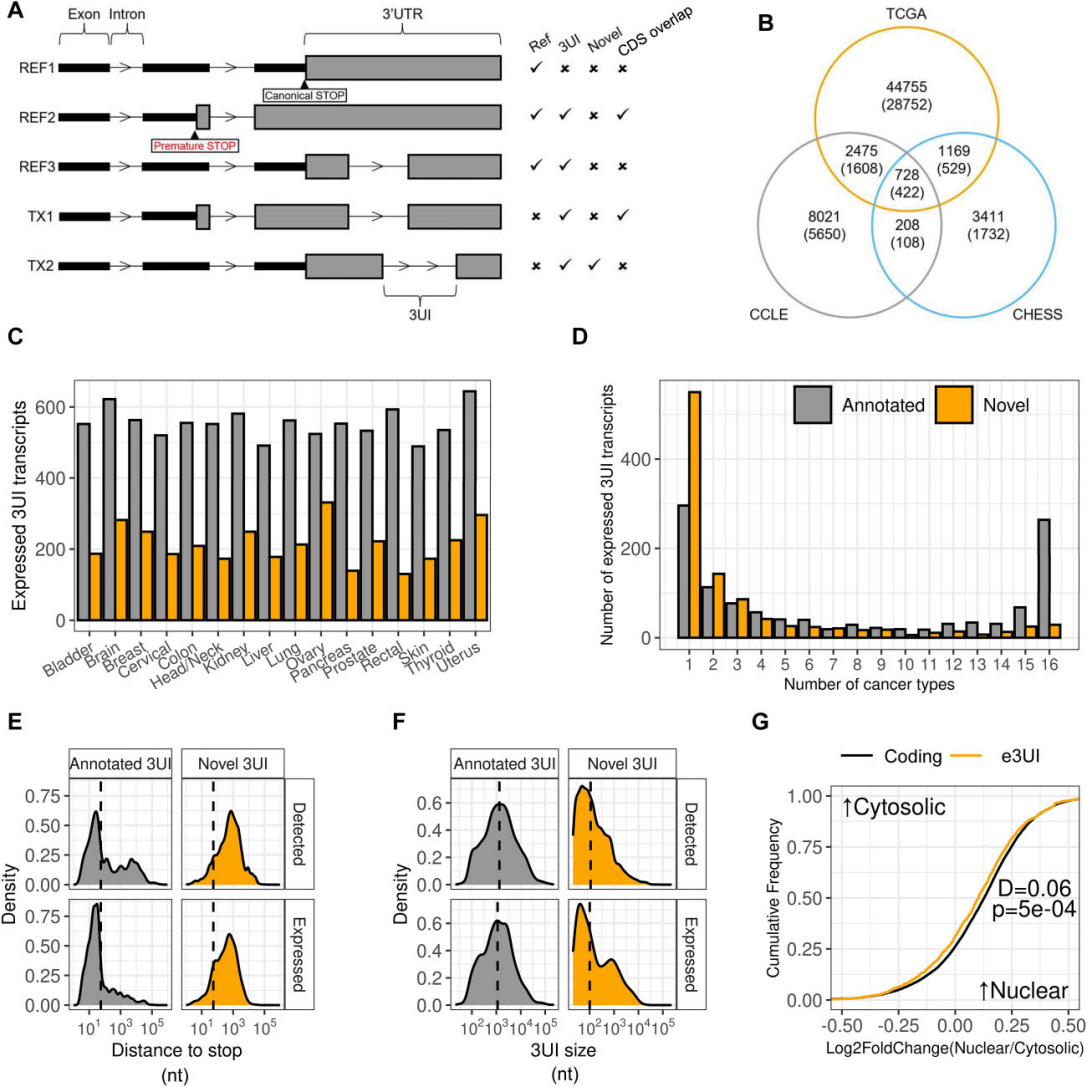
Detection and characterization of 3UI-containing transcripts. (**A**) Schematic diagram depicting the classifications of 3UI-containing transcripts. REF1 is a reference transcript lacking a 3UI, REF2 is a reference transcript that carries a CDS overlapping 3UI (co3UI) due to the introduction of an early PTC, REF3 is a e3UI-containing reference transcript, TX1 is a novel-3UI containing transcript which has a CDS exon- and intron-chain identical to that of REF2, TX2 is a novel-3UI containing transcript which has a CDS exon- and intron-chain identical to that of REF1. (**B**) Comparison between the total number of novel 3UI-containing transcripts detected in the TCGA assembly, CCLE assembly and CHESS (GTEx) assembly, numbers in brackets refer to e3UI transcripts. (**C**) Bar plot displaying the frequency that 3UI-containing transcripts are expressed in multiple cancer types. Transcripts are considered expressed where TPM > 1 and transcript/gene fraction > 0.25 in at least 10% of normal or cancer samples (see the “Materials and methods” section). Grey bars represent transcripts containing annotated 3UIs. Orange bars represent transcripts containing novel 3UIs. (**D**) Bar plot comparing the number of expressed 3UI-containing transcripts between different tissues. Grey bars represent transcripts containing annotated 3UIs. Orange bars represent transcripts containing novel 3UIs. (**E**) Density plots comparing the distance between the start of the 3UI and stop codon (or maximal distance where there are multiple 3UIs) between annotated and novel 3UIs in expressed transcripts versus total detected. (**F**) Density plots comparing the size of 3UIs between annotated and novel 3UIs in expressed transcripts versus total detected. (**G**) Empirical cumulative distribution function plot comparing nuclear-cytosolic distribution of e3UIs compared to protein-coding transcripts in HCT116. Only transcripts expressed TPM > 1 were considered. Left shift represents more cytosolic localization, right shift represents more nuclear localization. Kolmogorov–Smirnov test was performed to produce the D-statistic and *P*-value, *P*< .05 is considered statistically significant.

Examining transcript annotations from Ensembl, we identified 8566 transcripts with introns after the stop codon (4.5% of the Ensembl annotation). We noticed that many of these transcripts were almost identical to a matching isoform; however they appeared to contain an early termination codon meaning that introns that would normally be in the coding region were now found after the stop codon and were annotated as within the 3′ UTR. To identify transcripts with *bona-fide* 3UIs (those that only ever occur in the 3′ UTR and never overlap the coding region), we filtered out transcripts where a putative 3UI had the same splice donor or splice acceptor location as an intron in the coding sequence (CDS) of another transcript. A schematic overview of our transcript classification is shown in Fig. 1A.

To gain a more expansive view of splicing in 3′ UTRs, we examined the CHESS transcript annotation which is derived from normal tissue samples from the GTEx dataset. As these transcripts do not have an annotated CDS, we compared them to a high confidence set of reference transcripts (see the “Materials and methods” section). As the quality of this geneset is critical to the success of this approach, as a reference we used a highly filtered Ensembl set (see the “Materials and methods” section). We identified transcripts that contained the full and unaltered CDS identical to that from a reference transcript. Any introns in these transcripts after the stop codon we called 3UIs. If no 3UI had splice donors or acceptors that are used by any coding transcript, we marked the transcript as “e3UI containing”. Finally, if the 3UI is found in no transcript in the reference annotation we labelled it a “Novel 3UI” (Fig. 1A). We identified 12 790 3UI-containing transcripts, 5516 of which had an intron chain different from an Ensembl transcript, and 8502 of which were e3UI containing transcripts (2791 not matching an Ensembl intron chain) and 948 transcripts contained 3UIs that were novel in the CHESS set (Fig. 1B and Supplementary Data 2 and 6). While we attempt to reduce the possibility of false positives due to miss-annotation of the stop codon by applying strict filtering criteria to our reference set, we cannot rule out the possibility that errors in the reference set lead to errors in our 3UI calls.

It has been well established that cancerous cells show dysregulated patterns of splicing. In order to identify more examples, we assembled transcripts from 7897 tissue samples from 16 solid tumour types in TCGA (Supplementary Data 1), and 348 samples from the Cancer Cell Line encyclopaedia (CCLE; see Data availability statement). Transcripts were filtered so that at least one sample expresses the transcript at >1 TPM and transcripts that were fragments of reference transcripts, overlapped only with the intron of a reference transcript, or did not overlap a reference gene were removed. We used the same approach as described above to identify 3UIs. After filtering we identified a set of 60 848 transcripts from TCGA (Supplementary Data 3; 49 127 transcripts with novel intron chains) and 22 137 transcripts from CCLE (Supplementary Data 4; 11 432 with novel intron chains) carrying 3UIs, 51 991 and 16 966 of which, respectively, were e3UI-containing (31 311 and 7 788 novel chains; Fig. 1B and Supplementary Data 7and 8). Notably, the majority of these 3UIs used the canonical donor-acceptor (GT-AG) splice site (97.7% of co3UIs and 74.3% of e3UIs; Supplementary Fig. S1A) and had splice-sites that showed increased conservation compared to surrounding sequence (Supplementary Fig. S1B). Exclusively UTR located introns were shorter and more GC rich than 3UIs that share splice sites with CDS introns (CDS Overlapping or co3UIs; Supplementary Fig. S1C and D). While e3UIs and co3UIs had similar median phyloP conservation scores, the e3UI set had a small tail of elements with a higher level of conservation (Supplementary Fig. S1E). We studied the completeness of our experiment by examining subsets of colon cancer samples, showing that in this cancer type, the total number of 3UI-containing transcripts identified began to saturate at 100 samples (Supplementary Fig. S2A). Since our combined TCGA assembly contains over 78× this number, we expect that we are approaching the limit of 3UI-containing transcripts that can be identified this way.

To guard against the possibility of generating spurious 3UI-containing transcripts, we simulated 60 RNA-seq experiments using the Ensembl reference and passed these through our pipeline. We found that a small number of novel 3UI-containing transcripts were assembled. However, the number of transcripts found began to saturate by 20 samples, suggesting we had identified the majority of these artifactual 3UI-transcripts (Supplementary Fig. S2B), which were subsequently excluded from further analysis.

We defined a threshold for transcripts being expressed as having a transcript expression level of at least 1 TPM and requiring that the transcript represented at least 25% of all transcript expression from a gene (25% Tx/G). Across all samples 6934 e3UI containing transcripts were expressed at this level in at least one sample. Applying these thresholds to samples from colon cancer, we found that many transcripts identified are expressed in only a single sample (Supplementary Fig. S3). However, 764 transcripts reached this level in at least 10% of normal or colon cancer samples, these transcripts we describe as “broadly expressed” (Supplementary Fig. S3 and Supplementary Data 9). Despite the overall larger number of novel transcripts, more transcripts with previously annotated introns were classed as broadly expressed, and this pattern held across all cancer types examined (Fig. 1C). Broadly expressed transcripts with novel introns were mostly only associated with one cancer type. By contrast, transcripts carrying previously annotated introns were broadly expressed in either a tissue-specific manner, or in all cancer types examined (Fig. 1D). Broadly expressed e3UIs were more likely to use the canonical splice site than all e3UIs (88% versus 74%; Supplementary Fig. S1A). We tested if broadly expressed 3UIs were enriched in particular GO terms, but found no significant enrichments.

We benchmarked our assembly using long-read RNA-seq data from the ENCODE project. Overall, 53% of the e3UIs from broadly expressed transcripts in our TCGA dataset overlapped with e3UIs in the ENCODE long read data. As the ENCODE long read data was derived from 264 libraries, none of which came from primary cancer patient samples, we compared ENCODE long-read data with 3UIs from transcripts expressed at >1 TPM in each of the three cell lines that are in common between the two datasets—the colon cancer cell line HCT116, the liver cancer cell line HepG2, and the breast cancer cell line MCF7. In total 73%, 71%, and 72% of such 3UIs containing transcripts in HCT116, HepG2, and MCF7 cells respectively overlapped with 3UIs in the ENCODE set for the same cell line.

We also selected a panel of events to confirm using RT-PCR (Supplementary Fig. S1F).

It is generally thought that transcripts with splice junctions >55 nt from the stop codon are sensitive to NMD. For previously annotated exclusively UTR junctions, most junctions were closer than 55 nt from the annotated stop codon. This was not the case for the novel junctions. These were on average substantially further from the stop codon than 55 nt, which would be expected to make them sensitive to NMD (Fig. 1E, top). Surprisingly, this was not substantially different for transcripts broadly expressed in colon cancer (Fig. 1E, bottom). This suggests that either these transcripts have some mechanism for avoiding NMD, or that these transcripts are so highly expressed that a large amount of transcript is present despite being subject to decay. Novel introns were also substantially shorter than annotated ones, including those which are broadly expressed in colon cancer (Fig. 1F). Finally, to determine whether these transcripts are exported from the nucleus normally, we compared expression in the cytoplasm and nucleoplasm of HCT116 cells. We found that not only are e3UI containing transcripts efficiently exported from the nucleus, but their nuclear:cytoplasmic ratios are slightly, but significantly, lower than for non-3UI containing protein coding transcripts (Fig. 1G).

## 3′ UTR splicing regulates NMD in unexpected ways

Whilst it is widely accepted that NMD acts as an RNA surveillance mechanism to reduce the expression of truncated mRNA species, its role in regulating *bona-fide* (exclusively UTR) 3UIs is less well defined. Given that UPF1 is necessary for NMD, we predicted that its expression levels would negatively correlate with the proportion of spliced reads across 3UIs. For each sample in the TCGA colon dataset we calculated an “average 3UI percent spliced out (PSO)” value per sample (see the “Materials and methods” section). We then correlated this with the expression of each gene in each sample (see the “Materials and methods” section) to produce a correlation coefficient for each gene.

**Figure 2.**
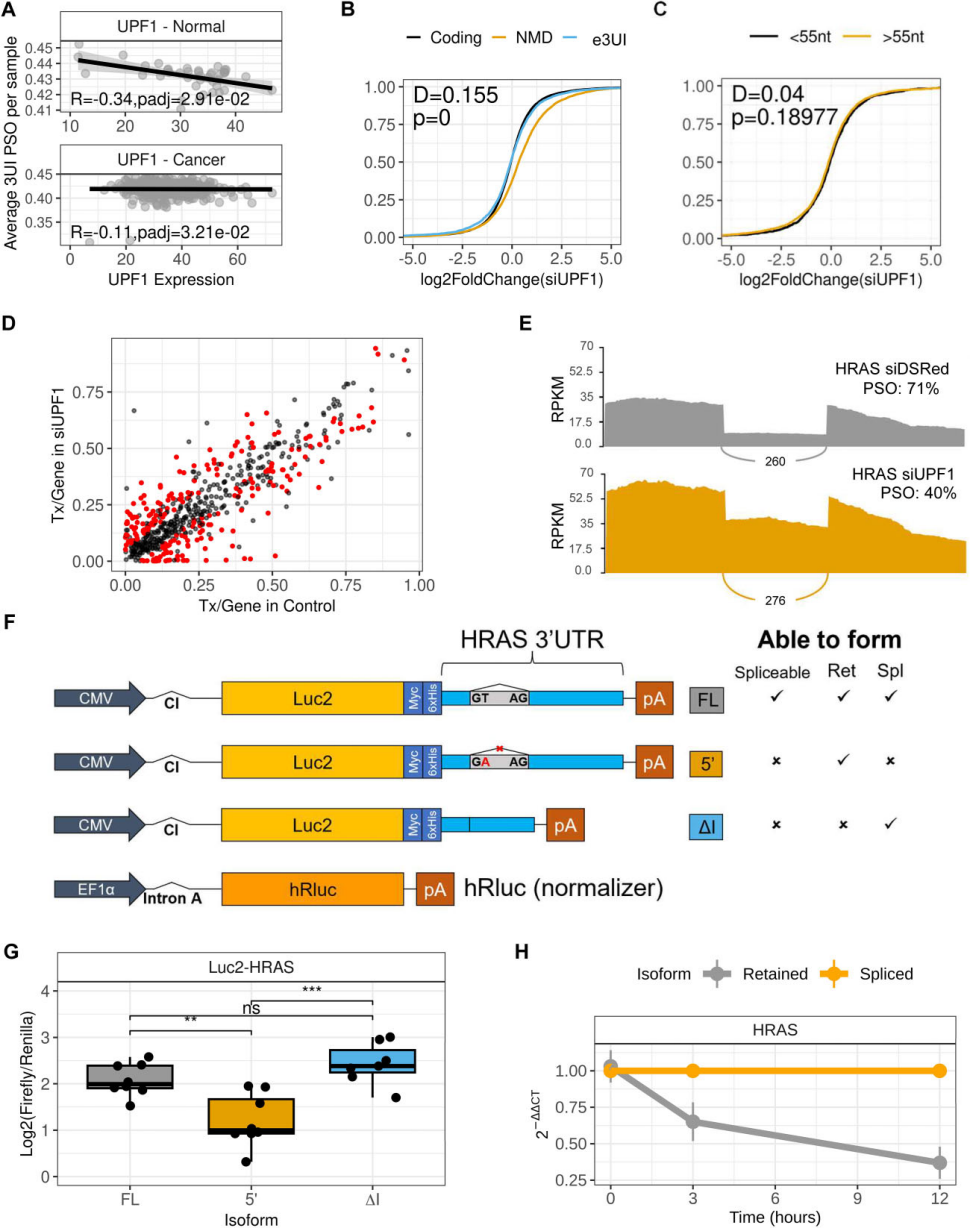
3UI splicing and retention regulates transcript sensitivity to NMD. (**A**) Correlation of UPF1 expression in each sample with its corresponding average 3UI PSO. Strength of correlation was determined by calculating Spearman’s rank correlation coefficient. (**B**, **C**) Empirical cumulative distribution function plots comparing the expression changes induced by UPF1 knockdown on: (**B**) transcripts that are protein coding, carry a e3UI, or are expected to be NMD sensitive (co3UI transcripts and transcripts annotated as NMD-sensitive); (**C**) transcripts that have their terminal 3UI more or less than 55 nucleotides from the termination codon; Kolmogorov–Smirnov test was performed, *P*< .05 is considered statistically significant. (**D**) DTU of 3UIs upon UPF1 knockdown. Each dot represents a 3UI-containing transcript. Red dots represent significant DTU (*P*< .05, effect-size > 5%). (**E**) Sashimi plot comparing retained versus spliced read coverage for HRAS in siUPF1 and negative control siDSRed. (**F**) Schematic diagram of Luciferase2-HRAS 3′ UTR reporter plasmids. Ticks and crosses indicate whether each construct is able to be spliced, and which isoform(s) it produces upon transfection. (**G**) Relative luminescence comparison between constructs upon transfection into HCT116 cells. (**H**) Relative stability of HRAS spliced and retained isoforms in HCT116 cells treated with actinomycin D measured by qPCR.

**Figure 3.**
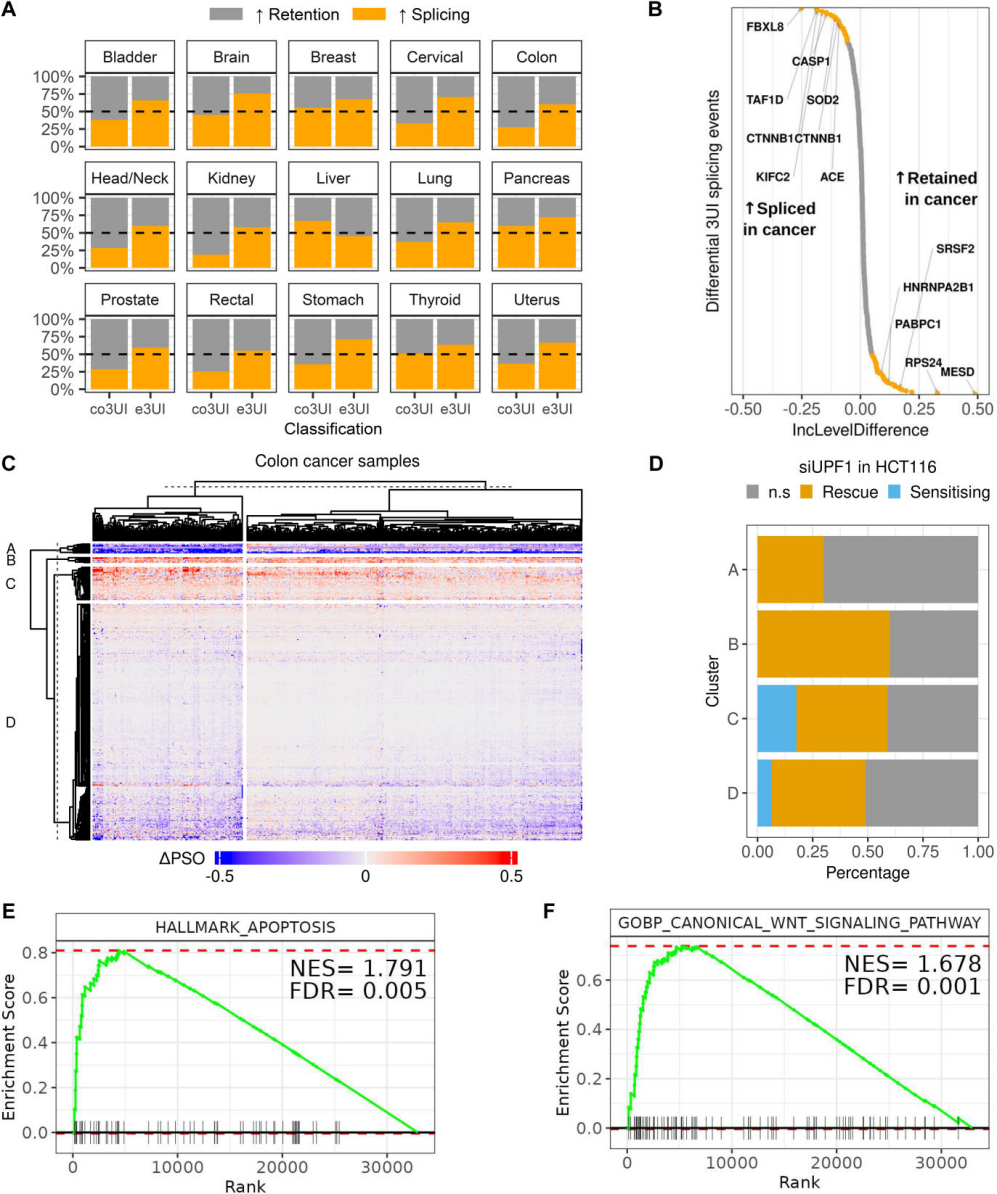
Widespread dysregulation of 3UI splicing in cancer. (**A**) Comparison of splicing of CDS overlapping 3UIs and exclusively UTR 3UIs between normal and cancer samples in a variety of tissue types from TCGA. Bar fill colour represent the percentage of transcripts that are either spliced more in cancer compared to normal or retained more in cancer compared to normal. (**B**) Distribution of significant intron inclusion events between normal and cancerous colon tissue. Grey dots represent significant events (padj < 0.05) with no effect-size threshold. Orange dots represent significant events with IncLevelDifference > 5%. Positive IncLevelDifference means more retention occurs in colon cancer (low PSO), negative values mean more splicing occurs in colon cancer (higher PSO). (**C**) K-means clustering of PSO values for events that were significantly different between normal and cancer samples. (**D**) Percentage of events in each cluster that were either less spliced (Rescue) or more spliced (Sensitizing) upon UPF1 knockdown. (**E**, **F**) GSEA for events which are differentially regulated between normal and colon cancer samples: (**E**) Apoptosis from the MSigDB Hallmarks Collection; (**F**) Canonical Wnt Signalling Pathway from MSigDB C5 Collection.

As expected, in normal samples we observed a negative correlation between UPF1 expression and average 3UI PSO for both e3UIs (Fig. 2A) and 3UIs that overlap protein coding introns in other transcripts (co3UIs; Supplementary Fig. S4B). Surprisingly, we observed a reduction of this correlation in cancer samples for e3UIs only compared to normal samples (r = −0.34 to r = 0.11, *P*= .001, Fig. 2A; Supplementary Fig. S4B). Such a difference between normal and cancer samples was also observed for other NMD components including UPF2, UPF3B, SMG1, SMG5, SMG6, SMG7, and SMG9 (Supplementary Figs S4A and C), and the difference was significant (*P*< .05) for UPF2, SMG1, and SMG7. Whilst SMG8 expression displayed no significant correlation with average 3UI PSO in normal samples, it displayed a positive correlation with the average e3UI PSO of the cancer samples and this difference was significant (*P*= .030). The only factor to show the opposite trend was UPF3A, and this difference was not significant. Such relationships may be difficult to interpret, due to feedback within the NMD pathway [46]. However, they do show that the relationship between splicing of e3UIs in cancer cells is different to that of either traditionally NMD activating co3UIs in cancer, or e3UIs in healthy samples.

To investigate this further we performed RNAi against UPF1 in HCT116 cells followed by RNA sequencing [Supplementary Fig. S5A and B and Supplementary Data 10 (Supplementary Tables S1–S3)]. UPF1 knockdown led to an increase in expression of traditional NMD targets, including transcripts labelled “Nonsense mediated decay” by Ensembl, and co3UI-containing transcripts from our assembly. However, this was not the case for transcripts with e3UIs (Fig. 2B and Supplementary Fig. S5C). Given that an intron has to be further than 55 nt from a stop codon to elicit NMD, we tested whether this result could be explained by the distance between stop codons and 5′ splice-sites. Surprisingly, we observed no significant difference between e3UI carrying transcripts where the intron was >55 or <55 nt from the stop codon (Fig. 2C). The same result is obtained if we use differential intron retention, rather than differential transcript expression (Supplementary Fig. S5D). These results confirm that co3UI-containing transcripts, which are likely to contain a protein truncating PTC, are indeed NMD sensitive; however, *bona-fide* e3UI-carrying transcripts may be NMD sensitive or insensitive, with equal numbers increasing or decreasing in expression (Fig. 2D) irrespective of their position relative to 55 nt after the stop codon.

Indeed, we find examples of splicing events that appear to rescue transcripts from NMD, such as in the oncogene HRAS, which carries an e3UI 8 nt from one stop codon, and 135 from another (Fig. 2E and Supplementary Fig. S5E). Through conducting both differential splicing analysis (Fig. 2E) and differential transcript expression analysis (Supplementary Fig. S5F) we reveal a significant increase in the expression of the 3UI retaining isoform in siUPF1 compared to a control siRNA, whilst no change is observed in the 3UI spliced isoform. We validated this result using qPCR with a second siRNA (Supplementary Fig. S5E). This was true irrespective of the inclusion or not of an earlier stop codon-containing cassette-exon in some isoforms (Supplementary Fig. S5F). This suggests that UPF1 negatively regulates expression of the 3UI-retaining isoform in colon cancer under nonknockdown conditions. This also suggests that splicing out the 3UI can offer protection from UPF1-mediated decay. To determine whether this effect is dependent on the physical act of splicing, we produced a set of Luciferase reporter constructs (Fig. 2F) which contained either: the full-length HRAS 3′ UTR (Luc2-HRAS-FL) where the 3UI can be spliced or retained; the HRAS 3′ UTR with a GT > GA 5′ splice site mutation (Luc2-HRAS-5′) where the 3UI is always retained; or the HRAS 3′ UTR with the 3UI cloned out (Luc2-HRAS-ΔI). We confirmed that the correct isoforms are produced upon transfection of each plasmid into HCT116 cells (Supplementary Fig. S5G). We observe that upon forcing 3UI retention there is a significant decrease in reporter activity, whilst removing the intron (via endogenous splicing of Luc2-HRAS-FL, or via cloning it out in Luc2-HRAS-ΔI) produces significantly more reporter activity (Fig. 2G). These results were replicated in a second colon cancer line - SW620 (Supplementary Fig. S5H). Given that this significant increase in reporter activity is observed in the Luc2-HRAS-ΔI construct which is unable to be spliced by the cell and therefore does not have an EJC upstream of the intron, we conclude that this phenomenon is likely EJC-independent. Further, measuring mRNA stability following transcription inhibition with actinomycin D reveals that the endogenous spliced isoform is more stable than its retaining partner (Fig. 2H). We also measured the effect of UPF1 knockdown on our luciferase reporters, finding that Luc-HRAS-FL and Luc2-HRAS-5′, but not Luc2-HRAS-ΔI was sensitive to UPF1 knockdown (Supplementary Fig. S5I). This suggests that the splicing event is removing destabilizing sequence elements present within the intron.

## 3′ UTR splicing is dysregulated in cancer

In order to investigate whether usage of 3UIs changes between normal and cancer samples, we used two complementary approaches: differential exon usage (DEU) and DTU. In this case, DEU measures the difference in usage of the retained intron that can be spliced out of the 3′ UTR. It has the advantage that it deals directly with the splicing event itself and is therefore closer to the mechanism. The disadvantages are: (i) It does not account for the rest of the transcript—e.g. does this splicing event change the total fraction of transcripts that have spliced 3′ UTRs or is splicing occurring in a transcript that already contains other 3UIs? (ii) An intron can be significantly changed in usage, but the transcript it is being spliced from might only represent a minor part of the output from the locus in question (e.g. splicing in a 5TPM transcript of a 100TPM gene). DTU solves these problems, but has its own drawbacks: (i) the same 3UI may be present in multiple isoforms, thus a change in usage of one 3UI containing isoform does not necessarily imply a change in the total 3UI usage; (ii) it is well known that global splicing is dysregulated in cancer, an increase in the number of splice isoforms used may lead to a decrease in the proportion of expression coming from any one isoform even if expression of the 3UI containing isoform itself is unchanged. Thus, we used both approaches to study cancer related changes in 3UI usage.

Firstly, by concentrating on the distribution of all changes (regardless of individual significance levels), across all 3UIs, most cancer types show a reduction of usage of the spliced isoforms in cancer for the majority of cases (Fig. 3A and Supplementary Fig. S6). This is consistent with a known increase in intron retention in cancer [47]. However, if we restrict our analysis to e3UIs, we see an increase in usage of the spliced isoforms in cancer for more transcripts than we see a reduction, in the majority of cancer types (Fig. 3A and Supplementary Fig. S6). In fact, for a given cancer type, there are many transcripts where there are no reads supporting the splicing of the intron in any noncancer samples, whereas splicing is supported in cancer samples. This suggests that *bona-fide* 3UIs behave in a qualitatively different way to introns that find themselves in the 3′ UTR by virtue of the inclusion of an upstream PTC, and that there is an increase in usage of transcripts containing *bona-fide* 3UIs in cancer. Indeed, we find that the difference observed between regulation of co3UIs and e3UIs is significant (^2^*P*-value <.001) for all 15 tissues tested.

Turning to individual examples of significantly changed 3UI PSO (FDR < 0.05), we see 123 introns with significantly increased splicing (i.e. decreased intron inclusion level) and 217 introns with significantly decreased splicing (i.e. increased inclusion level) in colon cancer (Fig. 3B). Of these 340 introns, 82 were contained within transcripts that also show significant DTU between cancer and normal conditions. Genes containing a 3UI with the largest increase in splicing include two different introns in the 3′ UTR of the gene encoding the WNT pathway regulator β-catenin (CTNNB1) and the inflammasome regulator Caspase-1 (CASP1). Genes containing a 3UI with the largest increase in retention in cancer include the gene encoding the poly-A binding protein PABPC1 and the WNT pathway regulator MESD. To account for potential differences in 3′ UTR splicing profiles within the population of colon cancer samples we conducted k-means clustering on these significant events (Fig. 3C). Clustering of samples revealed that a subset of the population had a greater degree of splicing/retention in either direction. Additionally, we did not observe any link between APC/CTNNB1 mutation status and splicing signatures. Clustering of events revealed four distinct clusters: (A) Lower splicing levels in cancer than normal; (B and C) Higher splicing in cancer; (D) Lower splicing levels in cancer, but with smaller effect sizes than cluster A, or a smaller number of samples affected. We predicted that events which are retained more in cancer would be NMD sensitizing, with intron retention acting as a potential mechanism to evade NMD. Likewise, we predicted that events with increases in splicing may be NMD rescuing. To test this we cross-referenced these events against our UPF1 knockdown in HCT116 (Fig. 2) and noted that whilst clusters B and C (e3UIs spliced more in cancer) do show an increased level of NMD rescue (i.e. decrease in proportion of spliced reads upon treatment with siUPF1), cluster A (retained more in cancer) does not show any selection of NMD sensitizing events (increase in proportion of spliced reads upon treatment with siUPF1), whilst cluster D only shows a small percentage (Fig. 3D). Together these results indicate that for events which are significantly different between normal and cancer samples, the introns which are spliced more in cancer (cluster B and C e3UIs) can reduce the overall NMD sensitivity of their host transcripts, whilst increased e3UI retention in cancer for cluster A and D e3UIs may increase the overall NMD sensitivity of these genes.

To identify potential functional implications of increased 3′ UTR splicing in colon cancer we ranked each splicing event from largest increase in splicing (lowest IncLevelDifference) to largest decrease in splicing (highest IncLevelDifference) and performed GSEA. When using the MSigDB Hallmarks collection we observed significant enrichment of gene sets related to apoptosis (Fig. 3E; NES = 1.791), DNA repair (NES = 1.735), IL2/STAT5 signalling (NES = 1.692), E2F targets (NES = 1.672), Wnt signalling targets (NES = 1.455), and Myc targets (NES = 1.441). Given that CTNNB1 harbours one of the most over spliced 3UIs between normal and colon cancer, and its function as a central regulator of the Wnt signalling pathway, we tested for enrichment of components of the canonical Wnt signalling pathway using the C5 (ontology) gene set and observed a significant enrichment (Fig. 3F; NES = 1.678, FDR = 0.001).

## 3′ UTR splicing modulates RNA–RBP and RNA–miRNA interactions

To investigate if 3′ UTR splicing may lead to the excision of binding sites for miRNAs and RBPs we used data from the ENCORI platform [40], which contains AGO-CLIP and CLIP-seq data. We found that the binding of a range of RBPs and miRNAs was enriched, in either all 3UIs, those that are broadly expressed in colon cancer, or those whose splicing significantly increases in cancer compared to normal (Supplementary Fig. S7). Enrichments were moderate (2–4-fold), but significant. Unsurprisingly various known splicing factors and spliceosomal components were enriched. The most enriched factor in both all, and e3UIs with increased splicing in cancer was TARBP2, a subunit of the RNA-induced silencing complex and binding of the m^6^A methylase FTO was also enriched in both broadly expressed and e3UIs with increased splicing in cancer. Additionally, we see significant enrichment of various miRNA–RNA interactions, with miR-324–3p being most significant (Supplementary Fig. S7B) in all e3UIs. We also observed a significant under-representation of several miRNAs in e3UIs over-spliced in cancer, with the most significant being miR-450a-5p (8-fold under-represented; Supplementary Fig. S7E). The enrichment of FTO prompted us to examine the presence of the m6A motif DRACH in our e3UIs. We found a significant enrichment of occurrences of this motif in both broadly expressed and over-spliced introns compared to all e3UIs (Supplementary Fig. S7F).

### Autoregulation of 3′ UTR splicing by the Wnt signalling pathway

Previous studies have shown that the Wnt signalling pathway is often dysregulated in colorectal cancer, in as many as 93% of samples [48], commonly leading to overactivation of CTNNB1, a central regulator of the canonical Wnt signalling pathway. CTNNB1 has two 3′ UTR-spliced isoforms, both use a common 5′ splice donor sequence, 15 nt from the stop codon, but have alternate 3′ splice acceptor sequences. Thus the isoform using the proximal 3′ splice acceptor contains a 305 nt long 3UI (short spliced isoform), whilst the isoform using the distal 3′ splice acceptor contains a 464nt long 3UI (long spliced isoform).

**Figure 4.**
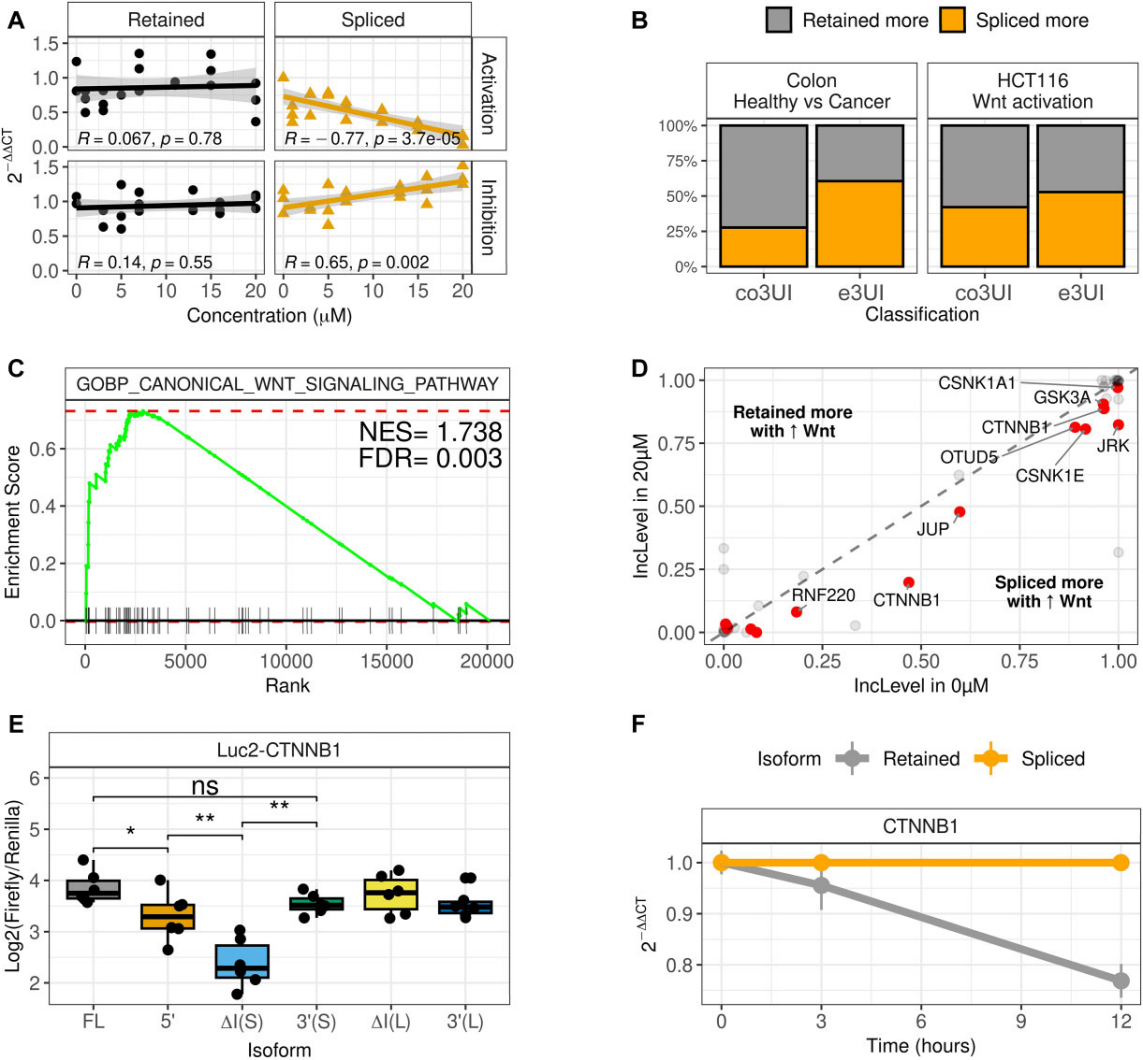
Wnt signalling regulates 3UI splicing of Wnt signalling pathway components. (**A**) Effect of increasing concentrations of CHIR99021 (Wnt signalling pathway activator) and IWR-1 (Wnt signalling pathway inhibitor) on expression of retained versus short-3UI spliced CTNNB1 isoforms in HCT116 cells. Pearson correlation coefficients and *P*-values are indicated. (**B**) Percentage of exclusively UTR and CDS overlapping 3UIs that are spliced more or retained more upon Wnt signalling activation in HCT116 cells, compared with normal versus colon cancer samples. (**C**) GSEA for events which are differentially regulated upon Wnt activation in HCT116 cells reveals enrichment of the canonical Wnt signalling pathway. (**D**) Differential intron inclusion of canonical Wnt pathway components in 0 versus 20 μM CHIR99021. Red dots represent significant events from RMATS (padj <0.05). (**E**) Relative luminescence comparison between Luciferase2-CTNNB1 constructs (Supplementary Fig. S8B) upon transfection into HCT116 cells. (**F**) Relative stability of CTNNB1 short-spliced and retained isoforms in HCT116 cells treated with actinomycin D.

Further to our finding that 3UI splicing is dysregulated in cancers generally (Fig. 3A) and specifically for CTNNB1 in colon cancer (Fig. 3B), we postulated that this could be in part due to hyperactive Wnt signalling. To test this we treated HCT116 cells with varying concentrations of CHIR99021, a GSK3β inhibitor, to cause accumulation of nuclear β-catenin protein and therefore activate the canonical Wnt signalling pathway. Isoform-specific qPCR revealed dose-dependent regulation of the short spliced isoform, which is the dominant isoform in HCT116 cells, with no effect on the retained isoform (Fig. 4A, top) and observed the opposite on treatment with IWR-1, which stabilizes Axin2 leading to β-catenin degradation (Fig. 4A, bottom). If this phenomenon was caused by AS, we would expect the expression of the retained isoform to displace the spliced isoform. However, this is not the case, therefore we suggest this is due to isoform-specific post-transcriptional regulation, e.g. regulation of mRNA stability. These results were similar in a second colon cancer cell line, SW620, and the human embryonic stem cell line, H9 (Supplementary Fig. S8A), with Wnt activation decreasing expression of the short intron spliced isoform relative to the retained isoform.

Next, we treated HCT116 with 20 μM CHIR99021 and conducted RNA sequencing. Splicing analysis revealed that roughly equal proportions of 3UIs were spliced more or retained more, compared with an increase in splicing for the majority of 3UIs in colon cancer [Fig. 4B and Supplementary Data 10 (Supplementary Table S4)]. Strikingly, we found that transcripts with increased 3UI splicing upon CHIR99021 treatment were strongly enriched in canonical Wnt signalling pathway components (NES = 1.738, FDR = 0.003; Fig. 4C) as also observed between normal and colon cancer samples (Fig. 3F). We also see an enrichment for the Wnt β-catenin Signalling Hallmarks collection, which mostly contains WNT pathway targets (NES = 1.4, pval = 0.04). A breakdown of the Wnt signalling pathway components regulated by CHIR99021 treatment can be found in Supplementary Fig. S9. Notably, the CTNNB1 long 3UI-spliced isoform, as well as a 3UI-spliced isoform of JUP (γ-catenin), were expressed significantly higher in CHIR99021 treated samples than untreated (Fig. 4D). From these results we suggest that dysregulation of 3UI splicing in colon cancer may be in part due to hyperactive Wnt signalling.

Finally, to elucidate the difference between each CTNNB1 3′ UTR isoform we created Luciferase reporter plasmids as before (Supplementary Fig. S8B and C). By mutating the 5′ splice site we observed a reduction in expression; however, when either of the 3′ splice sites are mutated this reduction is not observed (Fig. 4E). Additionally, we show that when the splicing of one isoform is prevented (through 3′ss mutation) that splicing of the other isoform is enhanced (Supplementary Fig. S8C), potentially explaining the lack of effect. Surprisingly, when the short intron is cloned out we observe a more drastic decrease in Luciferase expression, yet when the long isoform is removed (which encompasses the short isoform) this result is not replicated. These results were similar in SW620 cells, with short but not long intron removal leading to a significant decrease in Luciferase expression, although here the 5′ SS mutant construct had a slightly higher expression (<1.5× difference) than the full length construct (Supplementary Fig. S8D). This can be explained by either: (i) removing the short isoform produces a binding site for a destabilizing trans-factor, which cannot bind to the endogenously spliced isoform due to the presence of the EJC; (ii) the short intron contains stabilizing elements, but the long isoform contains additional destabilizing elements that mask/counteract these. These are outlined in Supplementary Fig. S10. Turning to the endogenous context in HCT116, we find that the 3UI-spliced isoform (short intron) is more stable than the 3UI-retaining isoform (Fig. 4F). We also see that the retained isoform, but neither of spliced isoforms increase in expression on UPF1 knockdown (Supplementary Fig. S8E). Together these results indicate that RNA stabilization may be dependent, at least in part, on the act of splicing itself, possibly due to regulation of the mRNP.

## Discussion

Here we have presented a novel transcriptome assembly (Supplementary Data 1) containing thousands of 3UI-containing transcript isoforms ranging from patient-specific transcripts to transcripts that are broadly expressed in many or all cancer types analysed. This assembly has allowed us to characterize the extent of 3′ UTR splicing in both normal and cancer samples and has revealed that 3′ UTR splicing regulates transcript expression in ways that may be both dependent and independent of the physical act of splicing itself in colon cancer. We make both individual splice event and transcript level quantification data against this assembly publically available (see Data availability statement).

During the production of this manuscript a parallel analysis of 3′ UTR splicing in TCGA data was published [49]. Chan *et al.* took a complementary approach to the transcript assembly approach taken here by identifying spliced reads that overlapped 3′ UTRs, but not any CDS annotations. Due to a lack of availability of the total dataset, we are unable to compare the transcripts we identified with the splice junctions they focused on. However, many of the findings are similar to ours at a global level. Our results complement and extend theirs in several key ways. Firstly, our approach allows a comparison of 3UIs that are always 3′ UTR located, with those that find themselves in a 3′ UTR due a premature stop codon. Secondly, we examine the effect of knocking down key NMD regulator UPF1 on 3UI expression at a global level. Thirdly, we show that not only do a large number of genes in the canonical WNT pathway appear to be regulated by 3UI splicing, but the activity of the pathway regulates 3UI splicing in turn.

Chan *et al.* find that the majority of their events of interest are closer than 55 nt to the stop codon, and suggest these events are therefore not NMD sensitive, demonstrating this for five example events. However, we find that many of our 3UIs are >55 nt from the stop codon, suggesting that by conventional wisdom, they should be NMD sensitive.

It has previously been shown that cancers can exploit NMD to promote pro-oncogenic cellular behaviours [50, 51] both through NMD-evasion by oncogenes [52] and NMD-sensitization of tumour-suppressor genes [53]. It has also been shown that NMD efficiency can differ between cell types during cell differentiation [54], and even between individual cells within a population [55]. Here we provide evidence that UPF1-mediated regulation of transcripts differs between those containing early, protein truncating PTCs compared to those which contain *bona-fide* e3UIs, at least in colon cancer. Additionally, we show that for *bona-fide* 3UIs, the location relative to the threshold of 55 nt past the stop codon has no significant effect on NMD sensitivity in colorectal carcinoma cell line HCT116. Given that EJCs would persist for 3UI events found >55 nt from the stop codon, whilst they would be removed from the mRNP by the ribosome for 3UI events found <55 nt, we suggest that any differences in UPF1 sensitivity between these 3UI containing transcripts are due to EJC-independent mechanisms.

NMD has also been shown to regulate the stability of transcripts with long 3′ UTRs (recently reviewed in [19]). Indeed, we do find that some 3UIs serve to protect the transcript from decay. In the case of HRAS, the spliced isoform is more stable (Fig. 2H) and the unspliced isoform, rather than the spliced isoform, is upregulated by UPF1 knockdown (Fig. 2E and Supplementary Fig. S5D and E). Splicing a 3UI could dramatically shorten the overall length of a 3′ UTR and rescue it from length-dependent decay. In HRAS the unspliced 3′ UTR is 260 nt long, whilst the spliced 3′ UTR is 152nt long. This suggests that UTR-length mediated regulation may be more widespread than previously considered. Alternatively, one mechanism by which UPF1 appears to regulate the stability of transcripts with long UTRs is through interactions with Ago2 and the miRNA pathway [56]. miRNAs primarily regulate transcript stability through binding to the 3′ UTR, and 3UI retaining isoforms could harbour more MREs, therefore the increased expression of 3UI-retaining isoforms (such as in HRAS) upon UPF1 knockdown could be explained by UPF1-Ago2-mediated regulation.

Whatever the source of the effect, it appears that it is not EJC-dependent, as deletion of the HRAS 3UI, which prevents EJC-deposition while also removing the intron sequence, has little effect on expression of a Luciferase reporter, while mutation of the 5′ SS, which would also cause a loss of EJC-deposition, but does not remove the intron sequence, causes a reduction in Luciferase expression (Fig. 2G) compared to the full length, spliceable HRAS 3′ UTR. Whether this form of UPF1-mediated regulation differs between noncancer and cancer, or between different cancer types, remains unclear.

In contrast, the effects of splicing in the 3′ UTR of CTNNB1 may be at least partially dependent on the act of splicing, as mutation of the 5′ SS and deletion of the short intron, at least in a Luciferase reporter, both cause a reduction in expression (Fig. 4E), although differences between the 5′ SS mutant construct, and the short intron deletion construct, neither of which can be spliced, and differences between cell-lines suggest further mechanisms are at play. These results differ from those of Chan *et al.*, who find that both mutation of the 5′ SS and deletion of the long intron increase expression of a Luciferase reporter (we find that deletion of the long intron has no significant effect on expression) [49]. These differences could be explained by the different models used (Colorectal carcinoma here, and Hepatocellular carcinoma in Chan *et al.*). Indeed, we find that the CTNNB1 generated by splicing the short intron is more used in Colorectal carcinoma samples than the isoform generated by splicing the long intron. However, our results do not provide support for the hypothesis that splicing in the 3′ UTR of CTNNB1 regulates expression by retention of the unspliced isoform in the nucleus via its interaction with U1, at least in HCT116 cells, as we would then not expect our 5′ ss mutant to reduce expression. Care must be taken interpreting the results of these reporter assays, as it is known that the effect of a 3′ UTR on transcript stability can be dependent on the identity of the CDS [57]. We did not believe it was appropriate to use a CTNNB1 open reading frame given the feedback we detect between Wnt pathway activity and 3UI splicing of Wnt pathway components. These results are supported by the results of the ActD stability experiment: the short spliced isoform is more stable than the retained isoform, and by the UPF1 knockdown RNA seq experiment, where only the retained isoform increases in expression on UPF1 knockdown (Supplementary Fig. S8E). These match the finding that the spliceable full length Luciferase reporter is more highly expressed than the unspliceable 5′ SS mutant reporter (Fig. 4F).

We propose a model for transcript stabilization by 3′ UTR splicing in colorectal carcinoma both by EJC-independent and EJC-dependent mechanisms (Supplementary Fig. S11). Regarding EJC-independent mechanisms, removal of 3UIs can remove destabilizing sequence elements such as MREs and DRACH motifs which leads to less miRNA binding and less m^6^A modification (note the enrichment of m^6^A demethylase FTO and DRACH motifs in 3UIs). Additionally, removing 3UIs likely plays a role in manipulating RNA secondary structure, which may impact RNA stability and translation [7], although a widespread study of 3′ UTR splicing on predicted RNA structure has not been conducted to date. By contrast, the effect of other e3UIs on transcripts may be dependent on the EJC. EJC deposition may protect local sequence from miRNA and RBP binding by steric interference, and does not always elicit NMD in colorectal carcinoma cells, as we have shown here. Additionally, it has recently been shown by two separate studies that EJC deposition appears to protect upstream and downstream sequence (∼100 nt) from m^6^A modification as part of a so-called “m^6^A exclusion zone” [58, 59]. This has been shown to be in part due to EJC core component EIF4A3 [58]. Hence splicing the 3′ UTR would not only remove potential m^6^A sites (via DRACH motif exclusion), but then further protect the surrounding sequence from modification, to increase RNA stability. Therefore we propose that, at least in the colorectal carcinoma setting, that splicing 3′ UTRs is able to modulate the composition of the mRNP to modulate post-transcriptional regulation beyond just regulating NMD-sensitivity.

We have also shown that manipulation of the Wnt signalling pathway regulates alternative 3UI splicing of canonical Wnt signalling pathway component transcripts. However, whether this is due to direct action of the Wnt signalling pathway (perhaps through kinase or phosphatase activity) or due to genes regulated by TCF/LEF transcription factors remains unclear. Nevertheless, understanding the molecular mechanisms downstream of Wnt hyperactivation in cancers remains critical to discovering novel therapeutic avenues. Wnt activating mutations in CTNNB1 or inactivating mutants in APC have been identified in 80% of colorectal carcinoma samples from the TCGA cohort studied here [48], but Wnt signalling is also commonly hyperactivated in many other cancers [60, 61] such as hepatocellular carcinoma [62], pancreatic cancer [63], and esophageal squamous cell carcinoma [64, 65]. Increased Wnt signalling activity has been linked with “stem-like” behaviours including increased cell proliferation [66], therefore tight control of its action is crucial for tissue homeostasis, which is lost in cancers. It is possible that aberrant splicing regulation of Wnt components contributes to this phenomenon. Additionally, noncanonical functions of the Wnt signalling pathway, for example in planar cell polarity [67], and also additional functions of β-catenin, should be considered in the cancer setting given their roles in cell adhesion, migration and invasion [68, 69]. In this regard we also show that JUP (γ-catenin, also known as junction plakoglobin) 3′ UTR splicing is regulated by Wnt signalling. JUP has been shown to function in a compensatory manner with β-catenin at adherens junctions [70, 71].

We conclude that splicing in the 3′ UTR is not a rare occurrence, especially in colon cancer. 3′ UTR splicing events represent more than transcriptional noise, and their inclusion or exclusion in different cellular contexts via AS represents a way to regulate transcript expression by multiple mechanisms, some of which may be EJC-independent.

## Supplementary Material

gkaf700_Supplemental_Files

## Data Availability

Raw sequencing datasets and corresponding salmon quantification files have been submitted to the NCBI Gene Expression Omnibus (GEO) under accession numbers GSE251665 and GSE251666. Transcript and gene quantifications, fraction expression calculations, exon counts, junction counts, PSIs, and differential splicing data for TCGA samples is available from Sheffield Online Research Data Archive (ORDA) under DOI 10.15131/shef.data.25612722. Custom pipelines and scripts (alongside software versions) used herein, are available at: https://github.com/sudlab/Cancer3UIs and Zenodo at the DOI 10.5281/zenodo.15790189.

## Notes

Present address: UCL Queen Square Motor Neuron Disease Centre, Department of Neuromuscular Diseases, UCL Queen Square Institute of Neurology, UCL, London WC1N 3BG, United Kingdom
