# Supplementary material for: Widespread 3′ UTR splicing regulates expression of oncogene transcripts through multiple mechanisms
Source: Nucleic Acids Res. 2025 Jul 26;53(14):gkaf700. doi: 10.1093/nar/gkaf700 (PMC13223760; doi:10.1093/nar/gkaf700)

Supplementary Figure 1. 3'UTR splice site conservation and examples. (A) Percentage of 3'UTR splice sites that utilize the canonical GT-AG splice site versus non-canonical sites for co3UIs, e3UIs and e3UIs which are broadly expressed in colon cancer. (B) Conservation of 5' and 3' splice site sequence compared to surrounding sequence. (C-E) comparison of co3UIs vs e3UIs: (C) 3UI length distribution, (D) GC content, (E) average conservation score. (F) RT-PCR using primers flanking the 3'UTR intron for HRAS, SRSF8 and CTNNB1, amplified from either HCT116 cDNA, HCT116 gDNA or No Template.

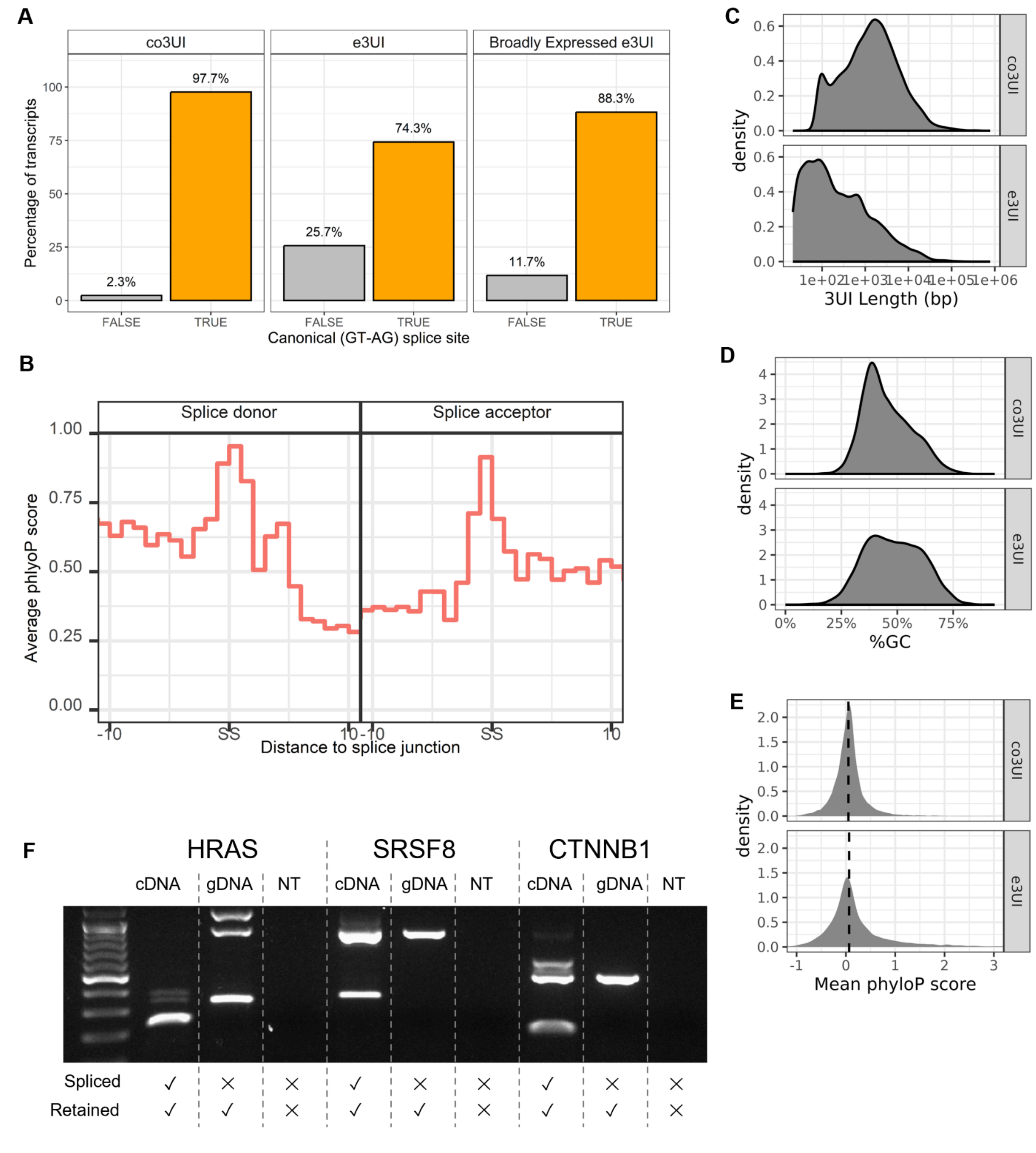

Supplementary Figure 2. Saturation analysis and artefact simulation. (A) Saturation analysis of 3UI detection utilising an increasing number of randomly sampled colon cancer RNA sequencing samples from TCGA shows detection begins to saturate at 100 samples. B) Number of novel 3UIs identified in 60 simulated RNA-seq datasets. Plot shows the number of transcripts and number of genes containing novel 3UIs for successively larger numbers of simulations

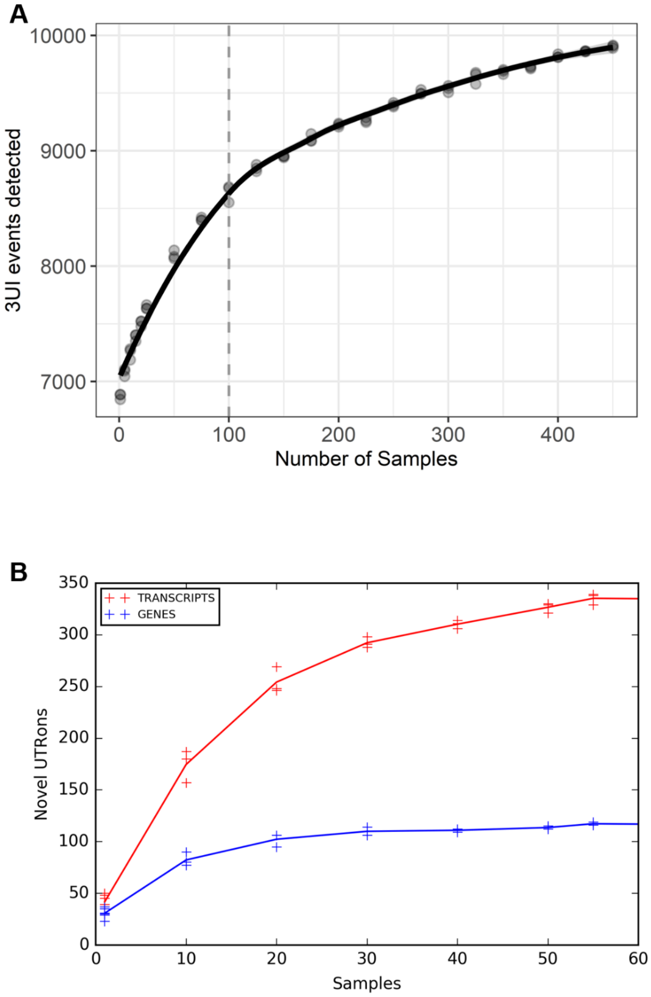

Supplementary Figure 3. Broad expression of 3UI-containing transcripts. Curve to represent the number of transcripts that meet each expression criteria in X% of colon cancer samples. Each colour represents an expression cutoff.

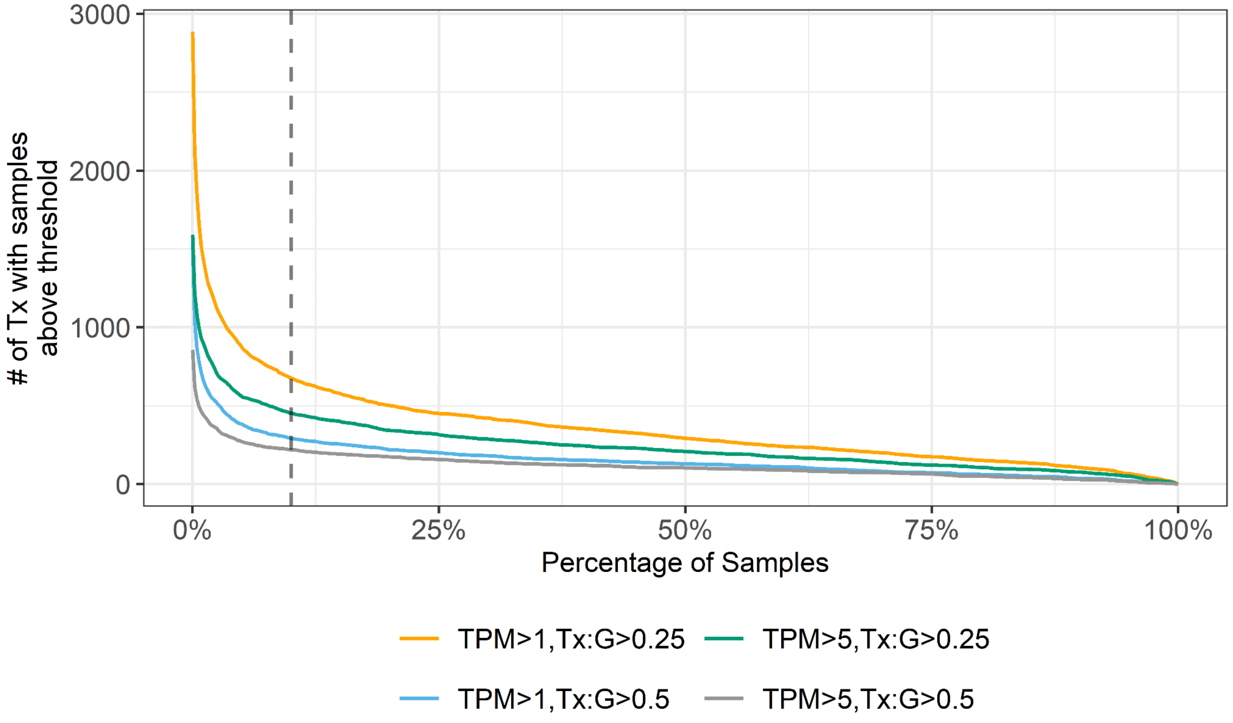

Supplementary Figure 4. Average 3UI PSO correlations. (A) Heatmap displaying the differences in correlation coefficient estimates between normal vs cancer and e3UI vs co3UI samples for NMD components. (B) Correlation of average 3UI PSO with normalised UPF1 expression. (C) Correlation of average 3UI PSO with normalised UPF3B expression. (D) Correlation of average 3UI PSO with normalised SMG8 expression.

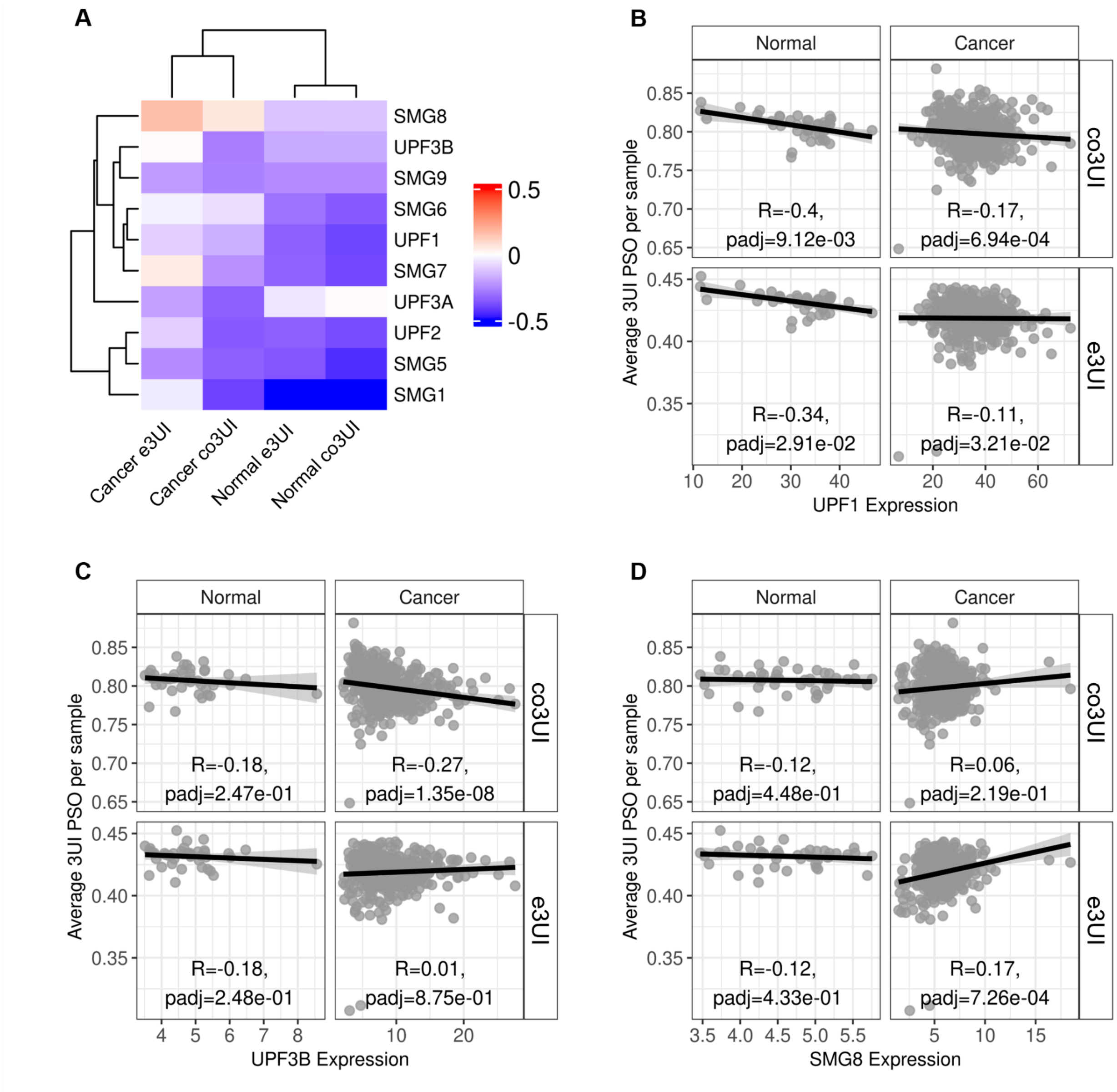

Supplementary Figure 5. UPF1 knockdown validation and effect on HRAS isoforms. (A) Western blot against UPF1 and Tubulin for HCT116 cells transfected with either siUPF1_1, siUPF1_2 or siDsRed (control). (B) RT-qPCR validation of UPF1 knockdown. (C) ECDF comparing the expression changes induced by UPF1 knockdown on transcripts with e3UIs or co3UIs. (D) ECDFs comparing the e3UI splicing changes induced by UPF1 knockdown that have their splice donor more or less than 55 nucleotides from the termination codon; Kolmogorov-Smirnov test was performed; (E) HRAS retained and spliced isoform expression measured by RT-qPCR following transfection with either siUPF1_2 or siDsRed (control). (F) HRAS retained and spliced isoforms (left) either with or without an upstream stop containing cassette exon and expression from RNAseq data following transfection with either siUPF1_1 or siDsRed (right). (G) Splicing of Luciferase2-HRAS 3'UTR plasmids upon transfection into HCT116 cells. FL produces both retained (R) and spliced (S) isoforms. ΔI produces only S. 5'ss mutant produces only R. (H) Relative luminescence comparison between Luciferase2-HRAS 3' UTR constructs upon transfection into SW620 cells. (I) Log2 ratio of firefly to renilla luciferase after co-transfection of HRAS plasmids with either siDsRed or siUPF1_2. Error bars represent 1 SEM. Values compared by t-test, n=4 bioreps, each the mean of two technical replicates. p-values shown above comparisons.

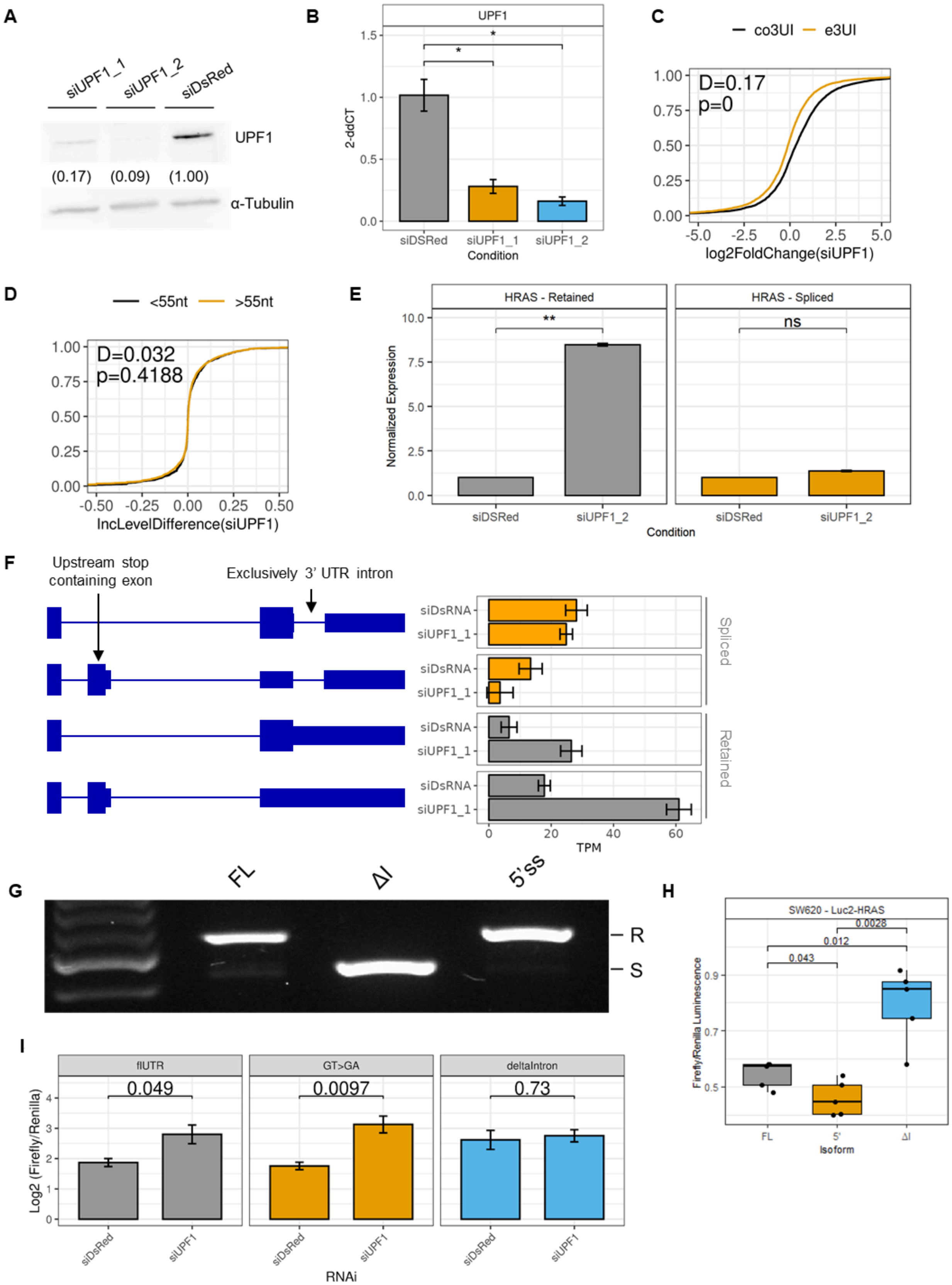
A
siUPF1_1
siUPF1_2
siDsRed
UPF1
(0.17)
(0.09)
(1.00)
α-Tubulin
B
UPF1
2-ddCT
siDSRed
siUPF1_1
siUPF1_2
Condition
C
co3UI
e3UI
D=0.17
p=0
log2FoldChange(siUPF1)
D
<55nt
>55nt
D=0.032
p=0.4188
IncLevelDifference(siUPF1)
E
HRAS - Retained
HRAS - Spliced
Normalized Expression
ns
siDSRed
siUPF1_2
Condition
F
Upstream stop containing exon
Exclusively 3' UTR intron
siDsRNA
siUPF1_1
Spliced
Retained
TPM
G
FL
ΔI
5'SS
R
S
H
SW620 - Luc2-HRAS
Firefly/Renilla Luminescence
0.0028
0.012
0.043
FL
5'
ΔI
Isoform
I
flUTR
GT>GA
deltaIntron
0.049
0.0097
0.73
Log2 (Firefly/Renilla)
siDsRed
siUPF1
RNAi

Supplementary Figure 6. Differential 3'UTR splicing between normal and cancer samples. Distribution of IncLevelDifferences for 3UIs between normal and cancer samples. Grey density plots represent e3UIs. Orange density plots represent co3UIs. x-axis truncated at |0.02| for easier interpretation.

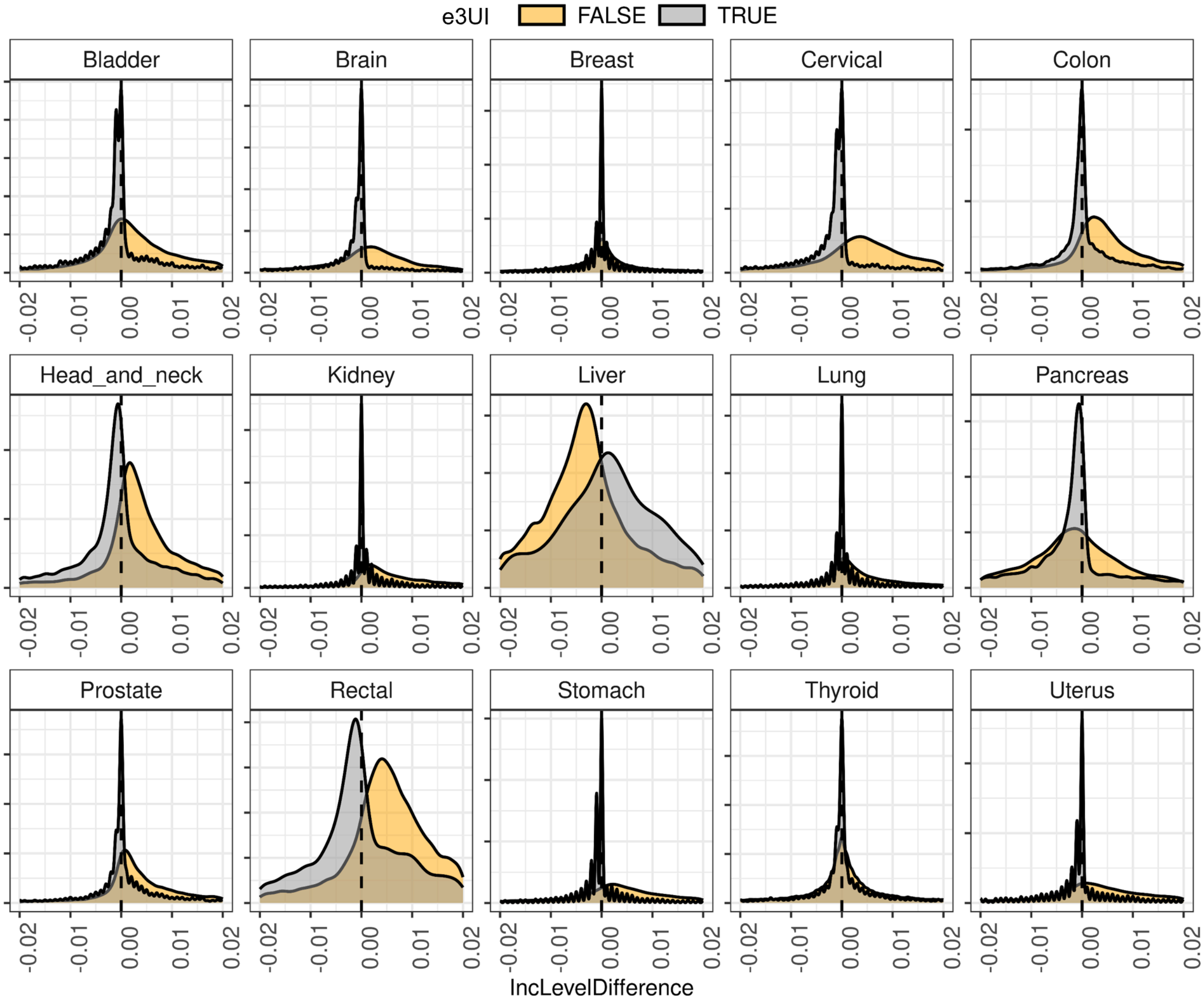

Supplementary Figure 7. RBP and miRNA enrichment analysis. (A-E) Enrichment analysis for 3' UTR intron sequences using CLIP or AGO-CLIP data. (A) RBPs in all detected e3UIs. (B) miRNAs in all detected e3UIs, supported by AGO-CLIP data. (C) RBPs in 3' UTR intron sequences from transcripts that are broadly expressed in colon cancer. (D) RBPs in sequences from 3' UTR introns that are spliced more in colon cancer than normal samples. (E) miRNA in sequences from 3' UTR introns that are spliced more in colon cancer than normal samples. (F) Number of m6a motif DRACH per e3UI in all e3UIs, e3UIs whose splicing increases in colon cancer, and e3UIs broadly expressed in colon cancer. ****=p<0.0001 wilcox test.

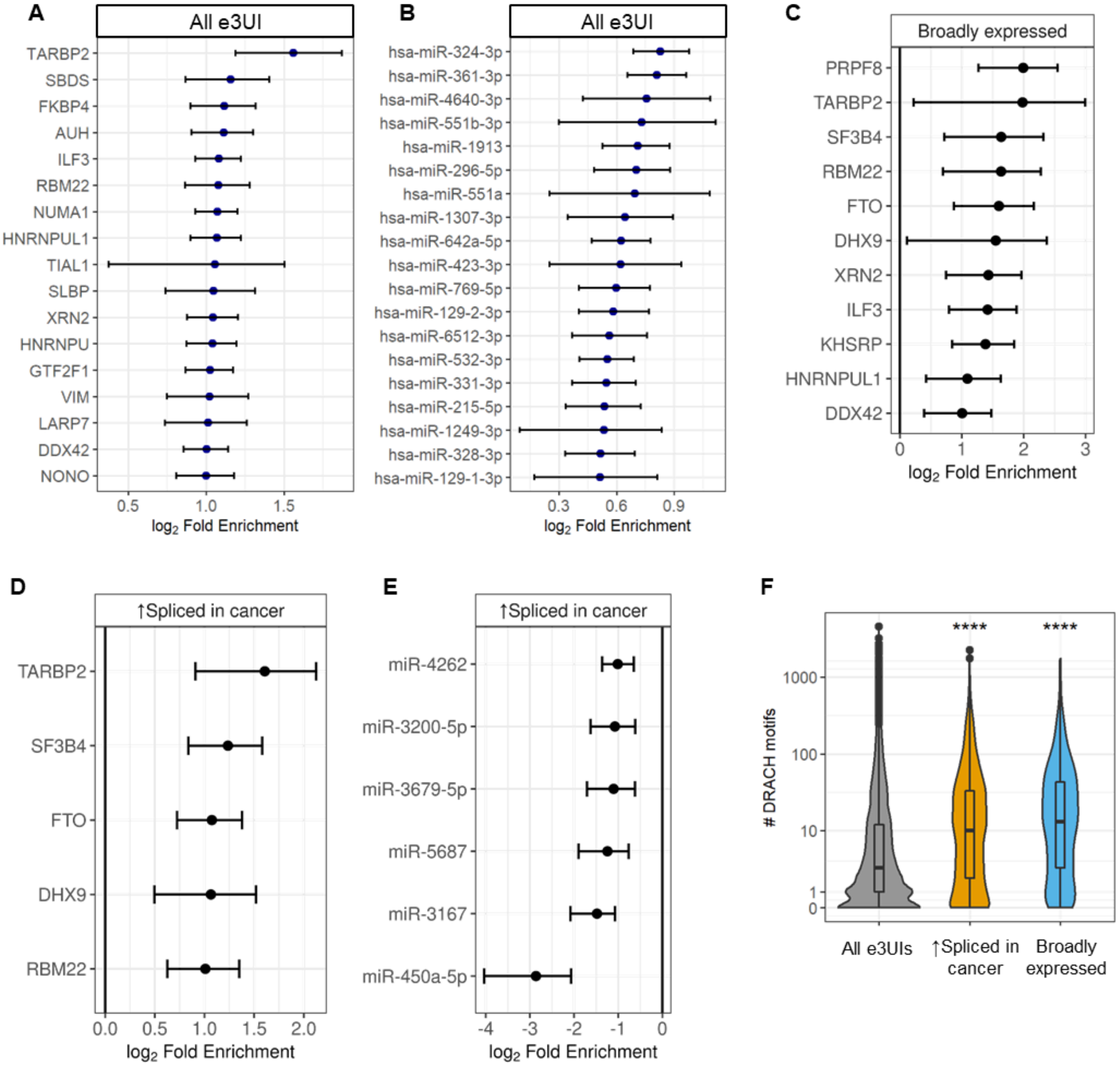

Supplementary Figure 8. Reporter assays in additional cell lines. (A) Comparison of Wnt manipulation assay conducted in H9 hESCs, HCT116 colorectal carcinoma cells, and SW620 colon cancer cells, measured by qPCR. n=3-4 biological replicates. Lines show least-squares regression line **(**B) Schematic representation of Luc2-CTNNB1 constructs, including splice site modification, and the isoforms they produce upon transfection. (C) Splicing of Luciferase2-CTNNB1 plasmids upon transfection into HCT116 cells. FL produces both retained (R) and short-spliced (S) isoform, long-spliced (L) is not visible. 5'ss mutant produces R only. ΔI(S) produces S only. ΔI(L) produces L only. 3'ss(S) produces R and L. 3'ss(L) produces R and S. (D) Luc2-CTNNB1 reporter assay conducted in SW620 colon cancer cells. (E) Expression of each CTNNB1 splice isoform upon knockdown of UPF1 vs control. Isoforms shown left, TPM from RNA-seq experiment shown right. Error bars = 1 SEM.

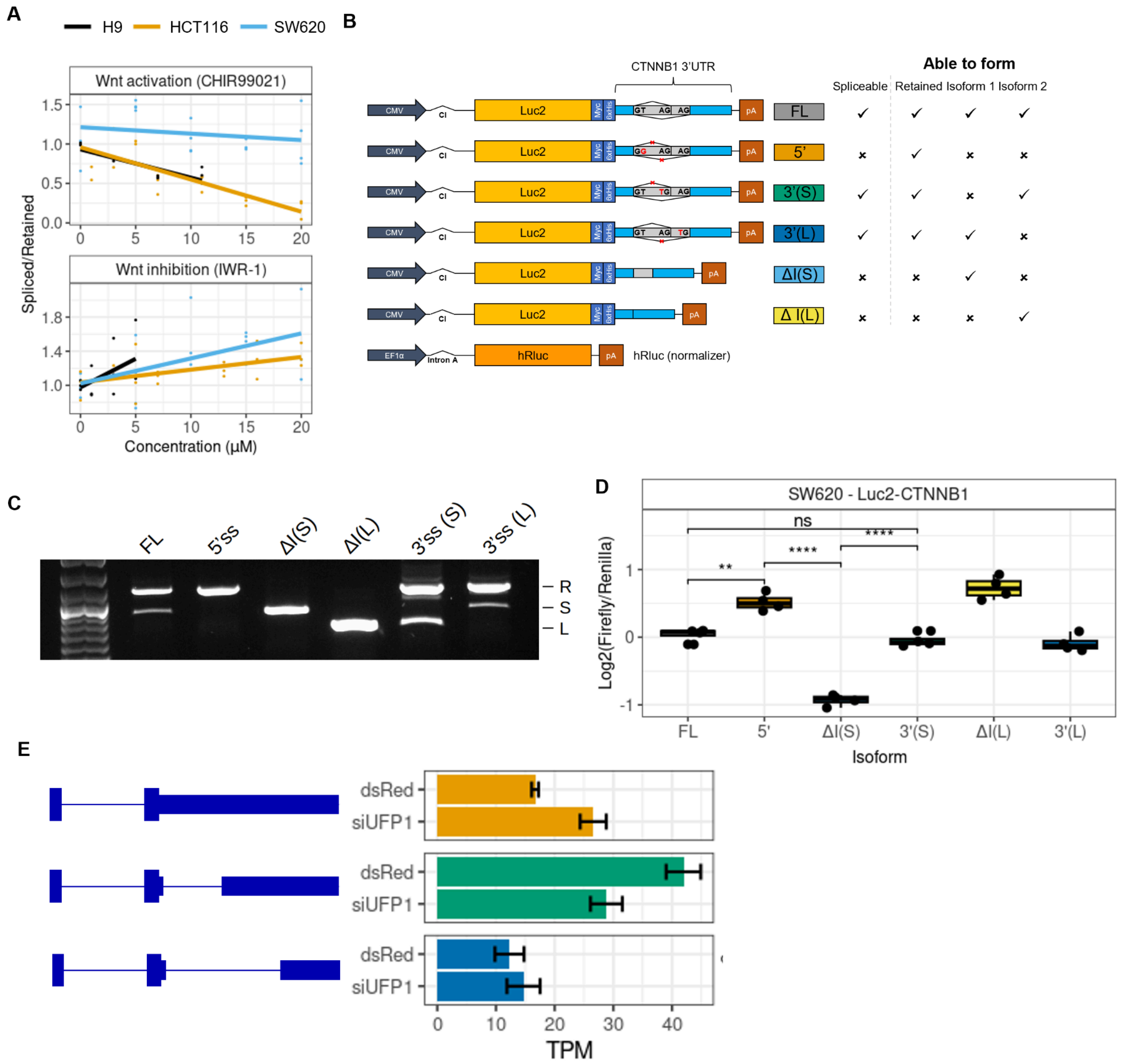

Supplementary Figure 9. 3'UTR splicing of the Canonical Wnt signalling pathway. Breakdown of the canonical Wnt signalling pathway colour-coded by whether each component 3'UTR is spliced more (orange) or retained more (blue) upon Wnt signalling activation in HCT116 cells. P-value derived from Gene Set Enrichment Analysis.

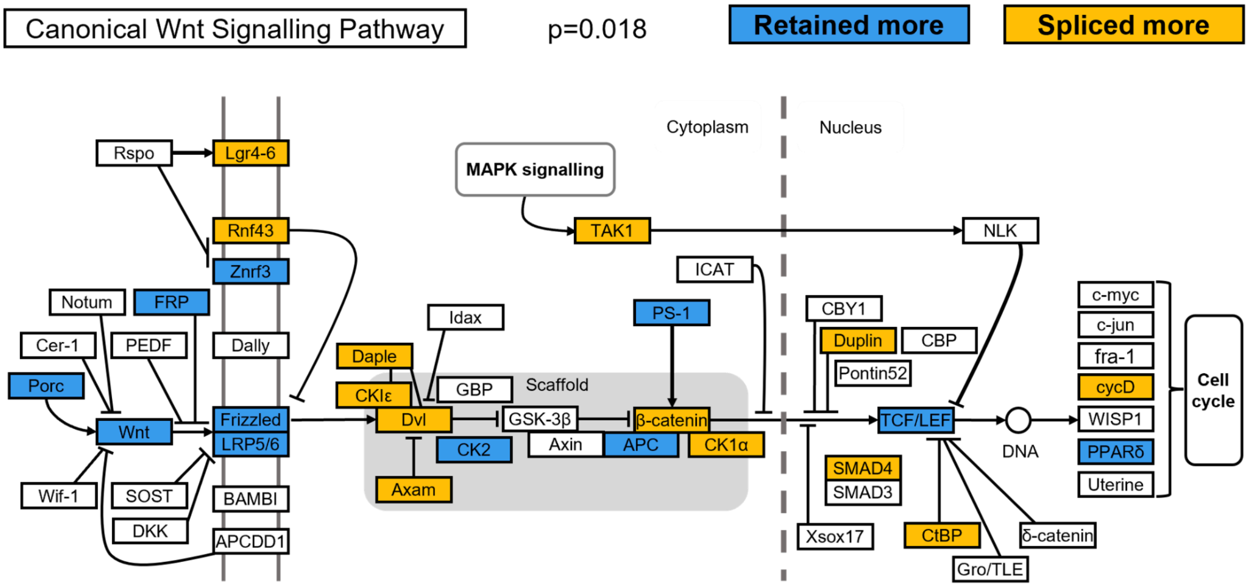

Supplementary Figure 10. Schematic model explaining Luciferase assay results. (A) Where the intron is cloned out via molecular cloning as opposed to splicing endogenously, no EJC will be deposited, therefore endogenous EJC-dependent regulatory pathways will impact full-length but not ΔI constructs. These may increase or decrease stability depending on the mRNP composition. (B) Through cloning out an intron we may introduce a cis-element which can bind trans-factors such as miRNAs or RBPs, which would otherwise be non-functional in the endogenous context due to the presence of the EJC.

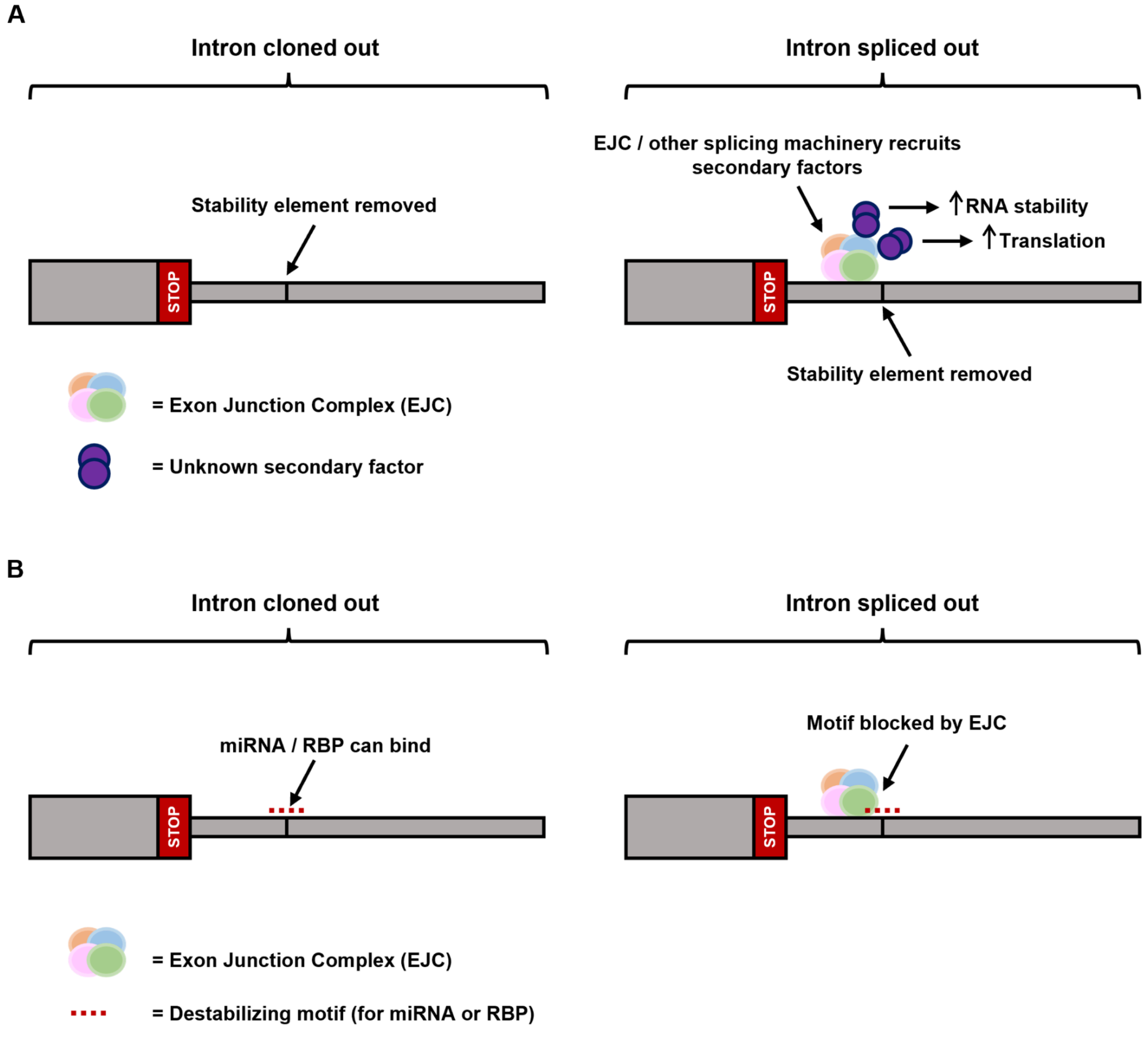

Supplementary Figure 11. Transcript stabilisation by 3'UTR splicing. Splicing 3'UTRs may remove sequences that would otherwise be subject to m6A modification, which is predominantly a destabilising marker. Additionally, the presence of the EJC upstream of the splice site prevents m6A deposition in the local vicinity, as part of a so-called "m6A exclusion zone".

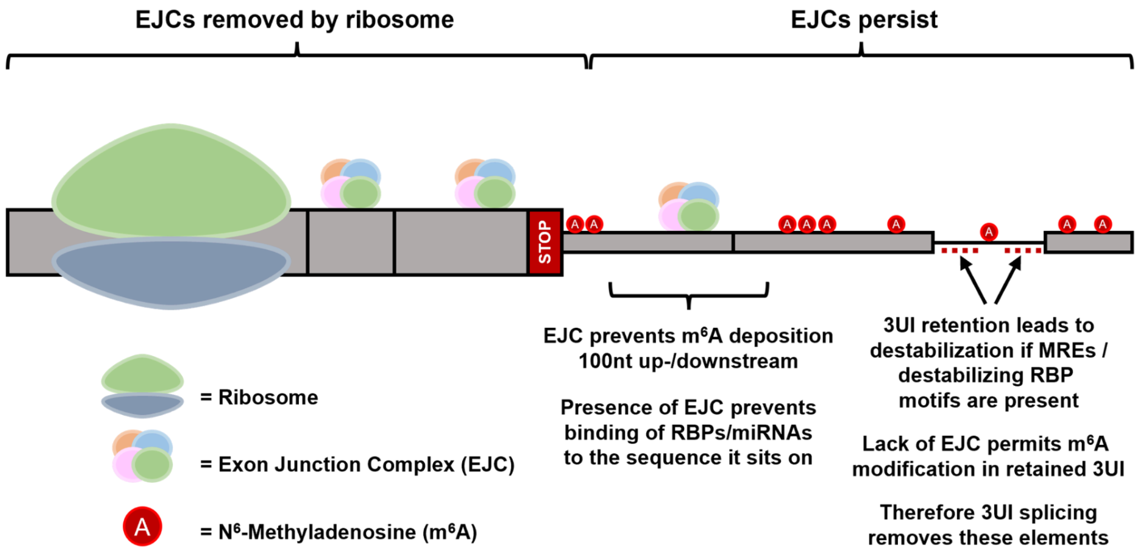

Supplement: gkaf700_Supplemental_Files [file gkaf700_supplemental_files.zip › Supplementary Figures.pdf]
